# *Clostridium perfringens* epsilon toxin induces blood brain barrier permeability via caveolae-dependent transcytosis and requires expression of MAL

**DOI:** 10.1371/journal.ppat.1008014

**Published:** 2019-11-08

**Authors:** Jennifer R. Linden, Claudia Flores, Eric F. Schmidt, Francisco A. Uzal, Adam O. Michel, Marissa Valenzuela, Sebastian Dobrow, Timothy Vartanian

**Affiliations:** 1 The Brain and Mind Research Institute and the Department of Neurology, Weill Cornell Medical College, New York, New York, United States of America; 2 Laboratory of Molecular Biology, The Rockefeller University, New York, New York, United States of America; 3 California Animal Health & Food Safety Laboratory System, San Bernardino Branch, University of California, Davis, San Bernardino, California, United States of America; 4 Laboratory of Comparative Pathology, Center of Comparative Medicine and Pathology, Memorial Sloan Kettering Cancer Center, The Rockefeller University, Weill Cornell Medicine, New York, New York, United States of America; University of Pittsburgh School of Medicine, UNITED STATES

## Abstract

*Clostridium perfringens* epsilon toxin (ETX) is responsible for causing the economically devastating disease, enterotoxaemia, in livestock. It is well accepted that ETX causes blood brain barrier (BBB) permeability, however the mechanisms involved in this process are not well understood. Using *in vivo* and *in vitro* methods, we determined that ETX causes BBB permeability in mice by increasing caveolae-dependent transcytosis in brain endothelial cells. When mice are intravenously injected with ETX, robust ETX binding is observed in the microvasculature of the central nervous system (CNS) with limited to no binding observed in the vasculature of peripheral organs, indicating that ETX specifically targets CNS endothelial cells. ETX binding to CNS microvasculature is dependent on MAL expression, as ETX binding to CNS microvasculature of MAL-deficient mice was not detected. ETX treatment also induces extravasation of molecular tracers including 376Da fluorescein salt, 60kDA serum albumin, 70kDa dextran, and 155kDA IgG. Importantly, ETX-induced BBB permeability requires expression of both MAL and caveolin-1, as mice deficient in MAL or caveolin-1 did not exhibit ETX-induced BBB permeability. Examination of primary murine brain endothelial cells revealed an increase in caveolae in ETX-treated cells, resulting in dynamin and lipid raft-dependent vacuolation without cell death. ETX-treatment also results in a rapid loss of EEA1 positive early endosomes and accumulation of large, RAB7-positive late endosomes and multivesicular bodies. Based on these results, we hypothesize that ETX binds to MAL on the apical surface of brain endothelial cells, causing recruitment of caveolin-1, triggering caveolae formation and internalization. Internalized caveolae fuse with early endosomes which traffic to late endosomes and multivesicular bodies. We believe that these multivesicular bodies fuse basally, releasing their contents into the brain parenchyma.

## Author summary

*Clostridium perfringens* epsilon toxin (ETX) is an extremely lethal bacterial toxin known to cause a devastating disease in livestock animals and may be a possible cause of multiple sclerosis in humans. ETX is well known to cause disruption of the blood-brain barrier (BBB), a critical structure necessary for proper brain function. Deterioration of this barrier allows entry of toxic blood-borne material to enter the brain. Although ETX-induced BBB dysfunction is well accepted, how this happens is unknown. Here, we demonstrate that ETX causes BBB permeability by inducing formation of cell-surface invaginations called caveolae in endothelial cells, the cells that line blood vessels. Importantly, only endothelial cells from the brain and other central nervous system organs appear to be a target of ETX, as the toxin only binds to blood vessels in these organs and not blood vessels from other organs. These ETX-induced caveolae fuse with other caveolae and specialized intracellular vesicles called endosomes. We predict that these endosomes engulf blood-borne material during their internalization, allowing material to travel from the blood, through the cell, and into brain tissue. We also show that expression of the protein MAL and caveolin-1 is necessary for ETX-induced BBB permeability.

## Introduction

Central nervous system (CNS) health is dependent on a carefully orchestrated homeostasis that ensures the optimal milieu for proper axonal conduction, synaptic transmission, and prevents infiltration of neuro-toxic substances[1–3]. To maintain this intricate environment, the CNS has developed a uniquely selective barrier, known as the blood brain barrier (BBB), which strictly regulates transfer of molecules from blood to the CNS, including harmful blood-borne material. Specialized brain endothelial cells (BEC) form the BBB and are structurally and functionally unique compared to endothelial cells found elsewhere in the body. This unique BEC phenotype is influenced by close contact with CNS pericytes, astrocytes, and neurons in a highly synchronized microenvironment called the neurovascular unit[4–8]. Together, these cells inhibit passage of blood-borne material by decreasing both paracellular and transcellular permeability. To inhibit paracellular permeability, adjacent BEC form multi-protein complexes called tight junctions (TJ) at cell-to-cell contacts, physically inhibiting diffusion around neighboring cells[9–11]. To inhibit transcellular permeability, BEC have developed specialized transport systems with very low pinocytic activity[12, 13]. Although there are descriptions of several different types of transcytotic pathways, only three have been definitively defined in brain endothelia. These include transport via macropinocytosis of non-selective cargo, receptor-mediated transport of clathrin-coated pits, and caveolae transport that can involve both ligand-specific internalization, adsorptive-mediated transcytosis, and fluid-phase transcytosis [13, 14]. An increase in BBB permeability, through either paracellular or transcellular mechanisms, can have devastating neuropathological outcomes and can even result in death. *C*. *perfringens* epsilon toxin (ETX) causes massive BBB permeability via unknown mechanisms. The goal of the work presented here is to define the molecular and cellular basis of ETX effects on the murine BBB [15, 16].

ETX is produced by *C*. *perfringens* toxinotypes B and D, and is responsible for causing the economically devastating and often fatal disease, enterotoxemia, in ruminant livestock [17–20]. These gram-positive, spore forming, anaerobic, bacteria can be found in the intestinal contents of healthy ruminant hosts as well as ubiquitously throughout the environment [16, 18, 21–28]. These toxinotypes are rarely detected in humans. However, ETX and the bacteria that produce it have been suggested to play a role in multiple sclerosis [29, 30]. Naturally occurring enterotoxmeia primarily affects young sheep and goats and is characterized by intestinal and pulmonary distress as well as severe CNS dysfunction including convulsions, tremors, spasms, and paralysis [15]. Exponential growth of *C*. *perfringens* type B or D in host gastrointestinal tracts results in secretion of a relatively inactive, 33kDA, epsilon protoxin (proETX). ProETX is enzymatically activated by host trypsin and α-chymotrypsin into a 29 kDA protein that is 1000 times more potent than its protoxin counterpart [16, 18, 31]. Once activated, ETX causes intestinal permeability via poorly understood mechanisms, allowing entry of the toxin into the bloodstream through unknown mechanisms[32–34]. Interestingly, ETX appears to target different organs depending on the afflicted species [32–41]. In ruminants such as sheep and goats, epsilon toxin appears to cause perivascular edema and interstitial edema in the lungs and myocardium as well as various forms of brain edema [20, 42–44]. Curiously, sheep appear to be more prone to ETX-induced brain edema compared to goats, and goats are more prone to ETX-induced colitis and severe diarrhea compared to sheep. In mice, ETX preferentially accumulates in the brain and kidneys, leading to BBB opening and occasionally tubular necrosis[20, 35, 36, 45]. Importantly, ETX-induced brain edema and BBB opening appears to be a common and relatively consistent pathological finding across species. Once in the brain, ETX is known to bind to brain vasculature and cause massive BBB permeability, allowing entry of ETX into the brain parenchyma [38, 39, 46–55]. Once in the parenchyma, ETX causes disastrous effects including demyelination and neuronal cell death[56–62]. However, the direct cellular targets of ETX versus indirect secondary injury are still controversial.

ETX-induced death and neurological symptoms in whole animals are believed to be a direct result of the toxin’s action on the CNS, most likely via massive perivascular edema[35–39]. However, it is possible that death could also be a result of pulmonary edema, often observed in ruminants [20, 42, 43]. Histopathological examination of brain tissue from naturally occurring and experimentally induced enterotoxaemia in ruminants most often reveals massive tissue damage including severe vasogenic edema and perivascular proteinaceous edema and less frequently, hemorrhagic foci and focal white matter necrosis [15, 19, 20, 42, 44, 45, 63–65]. These observations highlight the destructive effect ETX has on the BBB. ETX-induced BBB permeability happens within minutes to hours after ETX administration [38, 39, 46–54]. ETX’s effect on BEC has only been examined in a handful of publications but demonstrates a rapid increase in intraluminal albumin extravasation and internalization, as well as a rapid decrease in immuno-reactivity for endothelial barrier antigen (EBA), a marker for an intact BBB with unknown function [46, 49–51, 53, 66, 67]. Ultrastructure analysis of ETX-treated brains reveals vacuolated, electron-dense BEC with swollen mitochondria and attenuated cytoplasm [48]. Swelling of astrocytic end feet and an increase in aquaporin-4 expression, an important mediator of CNS water homeostasis, has also been reported [48, 67]. Although ETX-induced BBB permeability is a well-documented and accepted process that has been firmly established, the mechanisms involved in this process are poorly understood.

ETX may cause BBB permeability by increasing paracellular or transcellular permeability, or alternatively, by causing widespread endothelial cell death. As a member of the areolysin family of pore forming toxins, ETX is known to kill specific cell types. ETX-induced cytotoxicity is generally accepted to happen via formation of a heptameric pore which happens in a series of sequential steps [68]. Firstly, ETX binds to its receptor on the cell surface where it oligomerizes into a pre-pore complex. Cell death occurs after insertion of the pore complex into the cell membrane, resulting in a rapid decrease in transmembrane resistance and depletion of intracellular K^+^ Cl^-^, and ATP. This is followed by a slower intracellular increase in Na^+^ and Ca^2+^ [69–71]. ETX also causes mitochondrial membrane permeabilization and translocation of apoptotic inducing factor (AIF) to the nucleus [71]. Three proteins have been suggested as the ETX receptor, including the Hepatitis A Virus Cellular Receptor 1 (HAVCR1), the myelin and lymphocyte protein MAL, and caveolin-1 (CAV1) [62, 72–75]. MAL appears the most likely candidate, as it is the only protein demonstrated to be necessary for both ETX binding and cytotoxicity; however, this remains controversial.

The goal of this paper was to determine the cellular mechanisms of ETX-induced BBB permeability in mice with a specific focus on the roles of MAL and CAV1. Importantly, both of these proteins are expressed by BEC [76, 77]. We demonstrate that ETX specifically binds to the microvasculature of the CNS, and this binding requires expression of MAL, but not CAV1. Interestingly, expression of both MAL and CAV1 are necessary for ETX-induced BBB permeability. We further demonstrate that ETX treatment induces caveolae formation and elucidate the endocytic changes that lead to ETX induced BBB permeability in a murine model.

## Results

### Brain microvasculature is enriched in Mal and Cav1 expression

**Fig 1:**
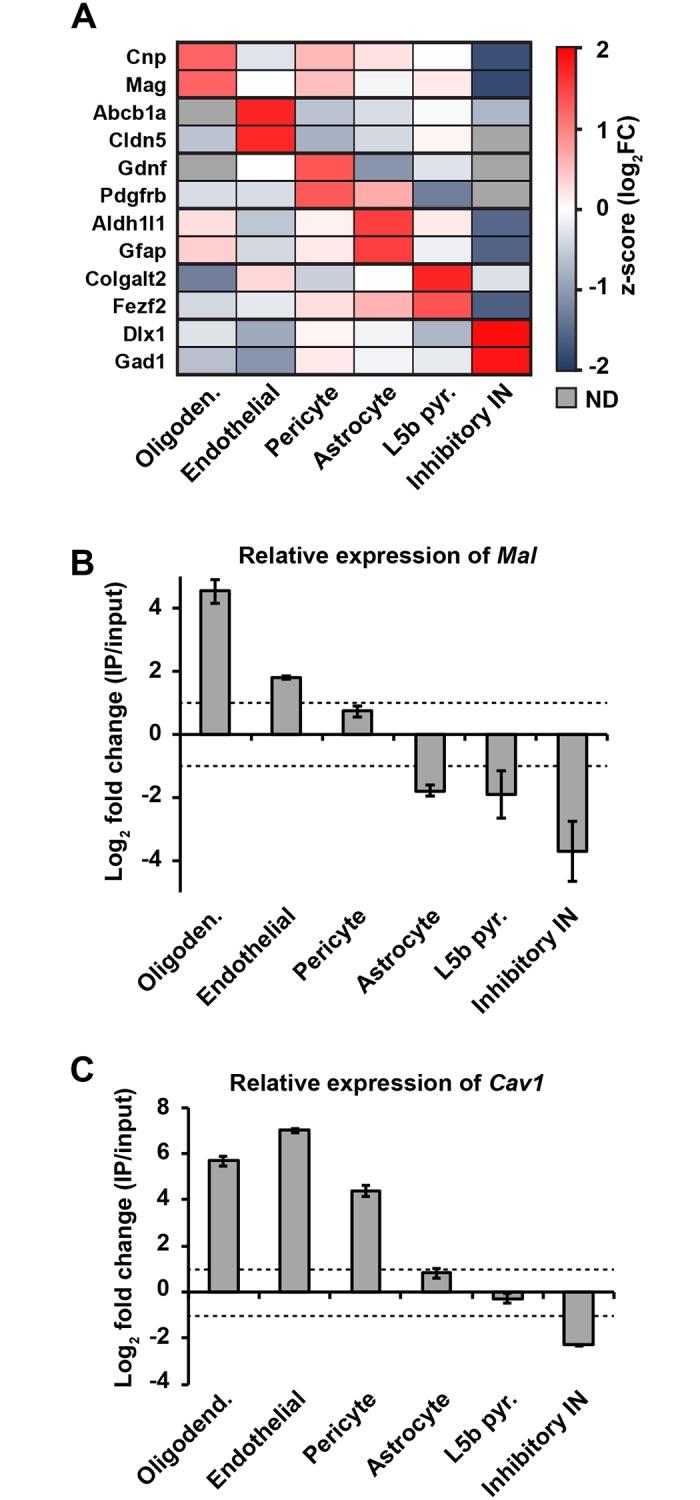
*Mal* and *Cav1* gene expression is enriched in brain endothelial cells. (A) Heatmap showing the enrichment of cell type marker genes in the TRAP IPs from oligodendrocytes, endothelial cells, pericytes, astrocytes, L5b pyramidal cells, and inhibitory interneurons compared to whole cortex input. Gray boxes indicate a failure to detect (ND) the gene in the data set. (B, C) Mean ± SEM of the ratio of *Mal* (B) and *Cav1* (C) expression (log_2_ fold change of RPKMs) in TRAP IP mRNAs compared to whole cortex input mRNAs in each of the CNS cell types from A. Dotted lines indicate 2-fold enrichment (positive value) or depletion (negative value).

Enriched *Mal* expression in murine CNS microvasculature has been previously demonstrated by comparing *Mal* gene expression in BEC versus liver and lung endothelial cells from TIE-2-GFP mice [76]. In addition, MAL expression in the murine CNS is limited to endothelial cells and oligodendrocytes based on RNA sequencing analysis[77]. Six transgenic mouse lines were used in which EGFP-tagged ribosomal protein L10a (EGFP-L10a) expression was targeted to distinct CNS cell types by cell-type specific bacterial artificial chromosome promoters including endothelial cells, pericytes, astrocytes, oligodendrocytes, L5b pyramidal cells, or inhibitory interneurons. The specificity of EGFP-L10a transgene expression in each cell type was confirmed by immunohistochemistry and the expression of cell-type specific molecular markers, as described previously[78–80]. Polysomes from each cell type were affinity purified from cortex by TRAP and polysome-bound mRNAs or mRNAs from whole tissue were analyzed by RNA sequencing. Fig 1A shows the enrichment of cell type markers in each of the TRAP data sets, demonstrating the specificity of the approach. Compared to whole cortex, *Mal* was highly enriched in oligodendrocytes and endothelial cells (Fig 1B). *Mal* was also expressed in pericytes, which, together with endothelial cells are critical constituents of the BBB. *Mal* was depleted from astrocytes, L5b pyramidal cells, and inhibitory interneurons (Fig 1B). Unfortunately, MAL protein expression in BEC could not be confirmed via IHC because there is no commercially available antibody that recognizes mouse MAL. *Cav1* expression in brain cell types was also evaluated using the TRAP method (Fig 1C). Compared to whole cortex, endothelial cells showed the highest relative expression of *Cav1*, followed by oligodendrocytes and pericytes. Taken together, these data indicate that Mal and Cav1 expression is enriched in CNS endothelial cells.

### ETX specifically targets CNS microvasculature and requires expression of MAL

**Fig 2:**
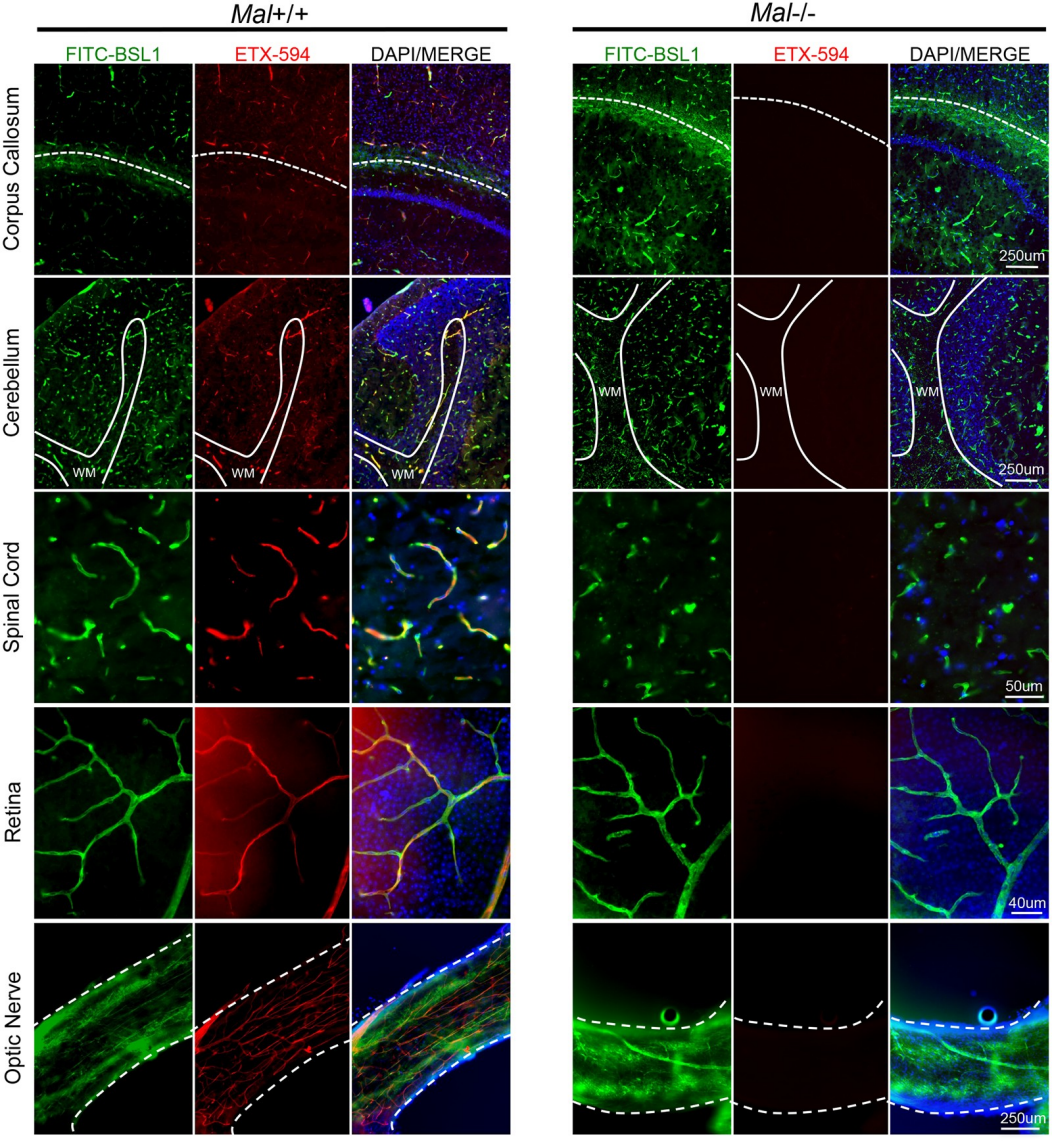
Epsilon toxin binds to the microvasculature of the CNS and requires expression of MAL. Wild type mice expressing *Mal+/+* or mice deficient in *Mal-/-* were intravenously injected with Alexa Fluor 594 conjugated ETX (ETX-594) for ten minutes then perfused with PBS to remove unbound toxin. ETX binding to microvasculature was evaluated in brain or spinal cord cryosections and whole mounts of retina and optic nerve. FITC-BSL1 was used to identify microvasculature. ETX-594 bound to the microvasculature of the brain including areas near the corpus callosum (white-dotted line) and the cerebellum (white matter tracts denoted by white line, WM) as well as the spinal cord (thoracic segment), retina and optic nerve (full thickness of optic nerve denoted by white dashed lines). ETX-594 binding was not detected in *Mal-/-* mice.

**Fig 3:**
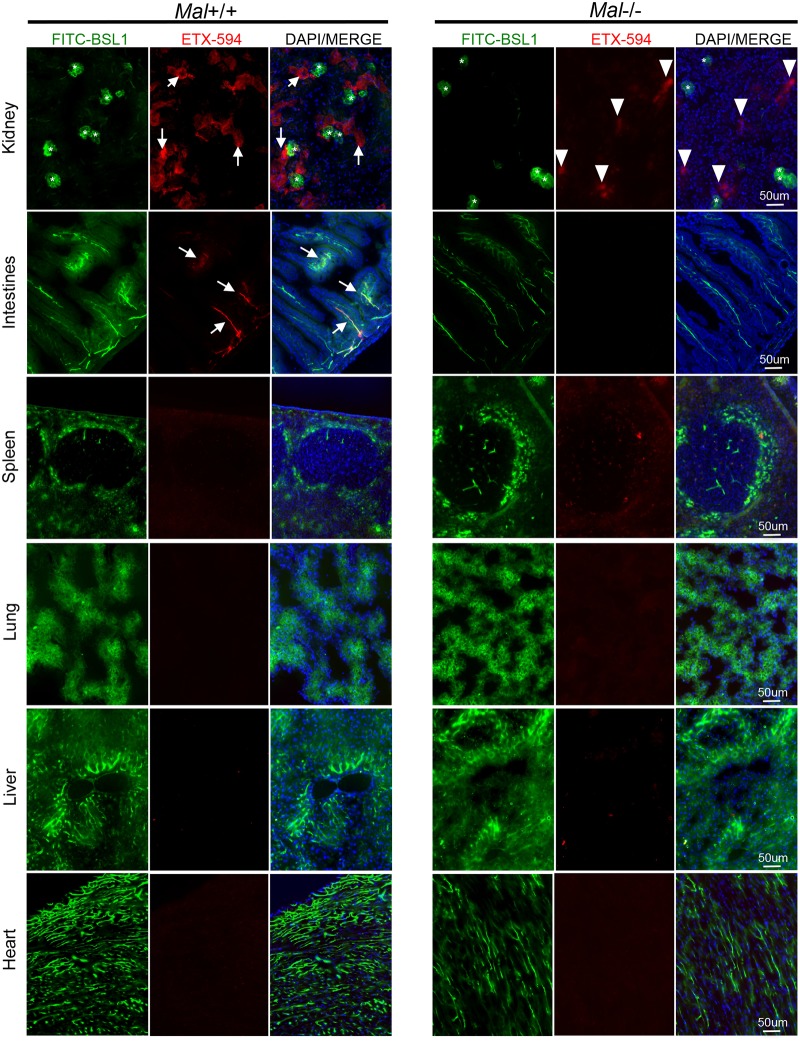
Epsilon toxin does not bind to the microvasculature of other peripheral organs. Wild type mice expressing *Mal+/+* or mice deficient in *Mal-/-* were intravenously injected ETX-594 for ten minutes then perfused with PBS to remove unbound toxin. ETX binding to microvasculature was evaluated in tissue cyrosections. FITC-BSL1 was used to visualize microvasculature. ETX bound to the epithelial cells of renal tubules of *Mal+/+* mice (white arrows) but not the glomerular capillaries (asterisks). In *Mal-/-* mice, ETX, can be seen accumulating in unidentified renal structures (white arrow heads). ETX is also observed binding to the microvasculature of the intestines in *Mal+/+* mice (white arrows), but not *Mal-/-* mice. ETX binding was not observed in the spleen, lung, liver or heart of *Mal+/+* or *Mal-/-* animals.

ETX is known to cause BBB permeability and preferentially accumulate in brains of ETX-treated mice. Because of the unique phenotype of BEC and ETX’s propensity to accumulate in brain tissue, we sought to determine if ETX has a unique affinity for murine CNS microvasculature compared to the vasculature of peripheral organs. Wild type mice were injected with Alexa Flour 594-conjugated ETX (ETX-594) for ten minutes and then perfused with PBS to remove unbound toxin from circulation. Microscopic evaluation of organ crysosections revealed that ETX preferentially bound to the microvasculature of all CNS organs including the brain, spinal cord, retina, and optic nerve (Fig 2). Importantly, ETX has been observed binding to both cervical and thoracic segments of the spinal cord; lumbar regions have not been evaluated. FITC-conjugated BSL1 (FITC-BSL1) was used to identify vasculature. Importantly, ETX was observed binding to all identified CNS microvasculature in these sections. ETX-594 extravasation into the brain parenchyma was not observed at this time point. In comparison, ETX was not observed to bind to microvasculature of peripheral organs including the kidney, spleen, lung, liver, and heart (Fig 3). ETX binding was observed in intestinal microvasculature, confirming previous results [74]. However, ETX-594 appears to bind to a small percentage of intestinal microvasculature while the majority of CNS vessels appeared saturated with ETX-594. Although ETX binding to renal tubules was also confirmed [35, 37, 39, 81], ETX binding to renal microvasculature and glomerular capillaries was not observed, indicating that ETX does not bind to renal endothelial cells. This data indicates that ETX selectively binds to murine CNS endothelial cells, and a lesser degree, intestinal endothelial cells.

MAL has been identified as the most likely ETX receptor [62, 74, 75]. To determine if MAL is necessary for ETX binding to CNS microvasculature, mice deficient in MAL (*Mal*-/-)[82] were injected with ETX-594 for ten minutes and then perfused. ETX did not bind to the CNS or peripheral microvasculature of *Mal*-/- mice (Figs 2 and 3). This data indicates that MAL expression is necessary for ETX binding to CNS microvasculature.

**Fig 4:**
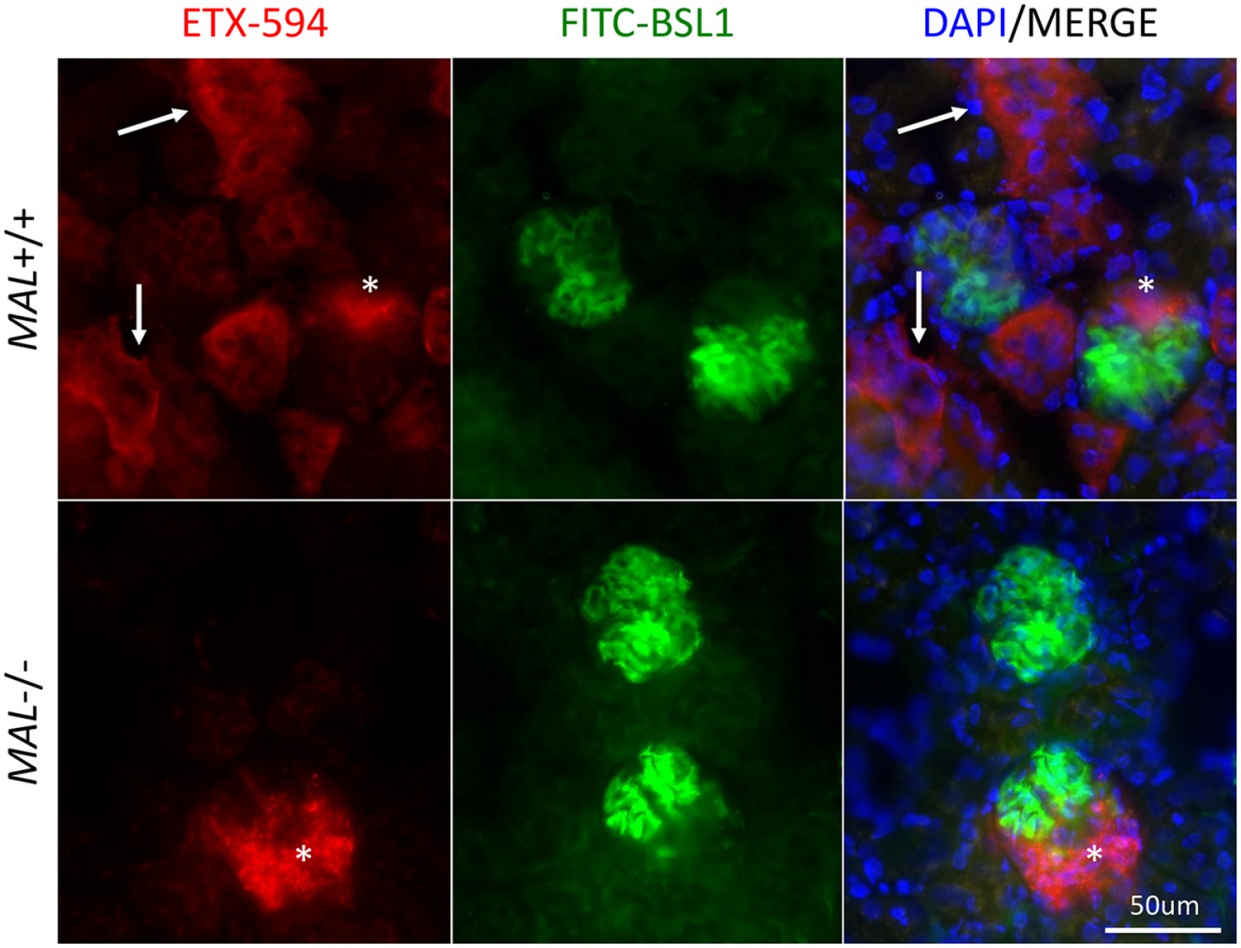
High magnification of *Mal+/+* and *Mal-/-* intravenously injected with ETX-594. Wild type mice expressing *Mal+/+* or mice deficient in *Mal-/-* were intravenously injected with Alexa Fluor 594 conjugated ETX (ETX-594) for ten minutes then perfused with PBS to remove unbound toxin. ETX binding to kidney tissue was evaluated by fluorescent microscopy. White arrows indicate ETX-594 binding to renal tubules. White asterisks indicate accumulation of ETX-594 in unknown renal compartment.

Interestingly, a small amount of ETX-594 was observed accumulating in unidentified regions of *Mal*-/- kidneys. Examination of renal sections at high magnification revealed two different ETX-594 binding patterns (Fig 4). One as a smooth, diffuse pattern on renal tubules and the other as punctate pattern in an unidentified renal compartment. Interestingly, both patterns are observed in *Mal+/+* mice, while the punctate patterns is only observed in *Mal-/-* mice. We speculate that the punctate ETX staining pattern my be a result of passive filtration of ETX from the bloodstream and not receptor mediated via MAL. In comparison, the smooth, diffuse ETX staining pattern to renal tubules appears to be MAL mediated as this pattern is present in *Mal+/+* mice and not *Mal-/* mice-.

### MAL is Required for ETX-induced BBB permeability

**Fig 5:**
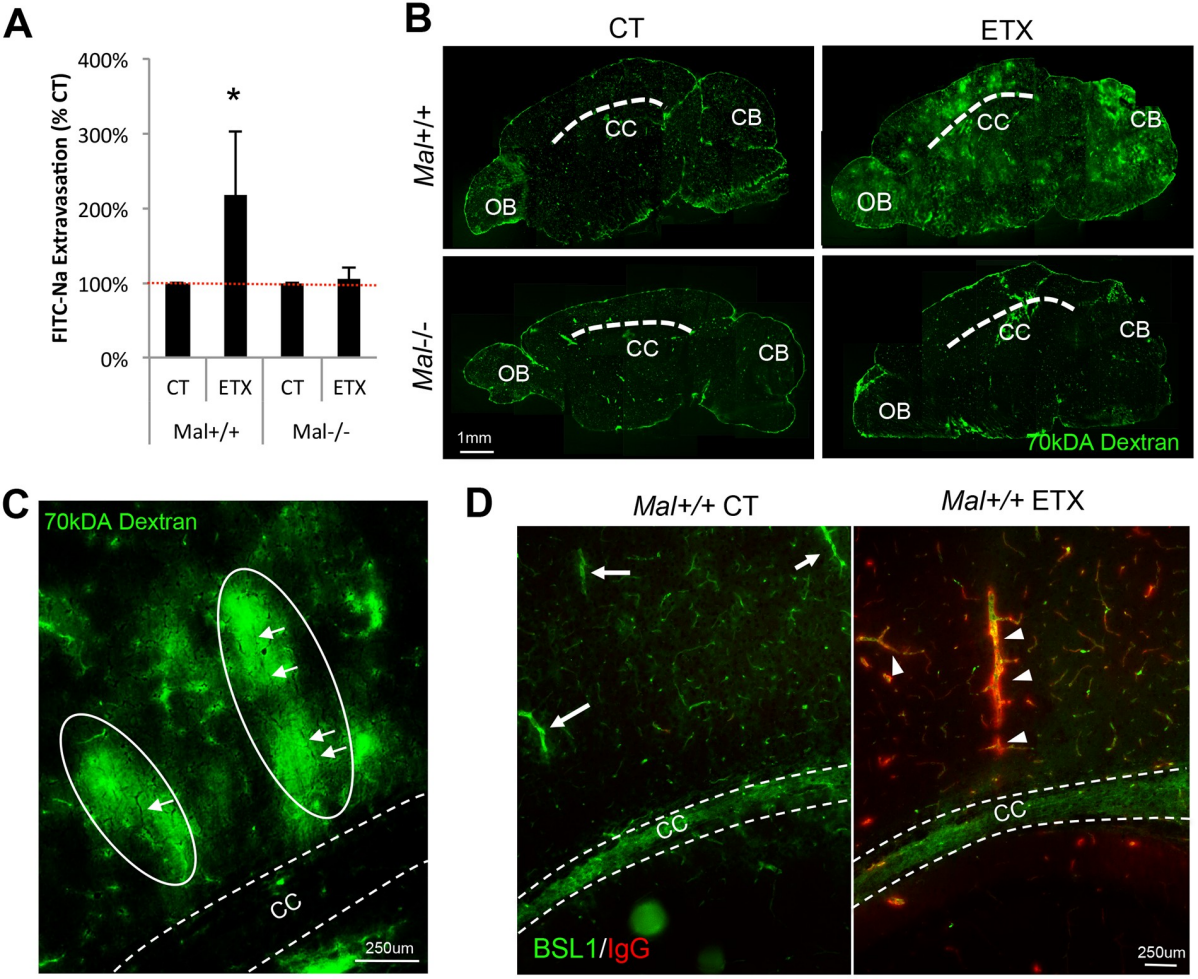
Expression of MAL is necessary for ETX induced BBB permeability. *Mal+/+* and *Mal-*/- mice were injected via IP with 5ng of active ETX per gram of body weight; saline treated animals were used as controls. After 1 hour, all animals were intravenously injected with FITC-Na for ten minutes then sacrificed. Brains were harvested and the amount of FITC-Na per gram of brain was calculated using spectrometry. Results are normalized to individual genotype controls and expressed as FITC-Na extravasation (% CT). Results expressed as Mean ± SEM, *p<0.05 determined by ANOVA, n = 3–5 mice per group. (B) Alternatively, ETX or saline treated *Mal+/+* and *Mal-/-* mice were intravenously injected with 70kDA FITC-dextran for ten minutes one our after treatment. Brains were harvested, cryoprotected, and sectioned. 70kDA FITC-dextran extravasation into the brain parenchyma was determined by fluorescent microscopy. Dashed line identifies corpus callosum (CC). OB, olfactory bulb. CB, Cerebellum. Sections are approximately 1 to 1.5 millimeters to the left of the mid-sagittal plane. (C) Higher magnification of pericallosal white matter in ETX treated *Mal+/+* mice identify ovoid shaped lesions (white ovals) perpendicular to corpus callosum (CC, width denoted with white dotted lines). White arrows point to shadows in FITC-dextran halos presumed to be central veins. (D) Sagittal sections of perfused control or ETX treated *Mal+/+* stained for endogenous mouse IgG (red). FITC-BSL1 (green) was used to identify microvasculature. Dashed lines identify width of corpus callosum (CC). In control treated mice, limited IgG extravasation was observed, even around large vessels (white arrows), presumed to be deep medullary veins. In ETX treated mice, IgG extravasation is very prominent, especially around large vessels (white arrowheads), presumed to be deep medullary veins.

Because MAL is necessary for ETX binding to CNS microvasculature, we next sought to determine if MAL expression was necessary for ETX-induced BBB permeability. *Mal*+/+ and *Mal*-/- mice were treated with 5ng of ETX per gram body weight for one hour, and BBB permeability was evaluated by extravasation of various molecular tracers. Mice treated with saline were used as controls. In select experiments, control and ETX treated mice were intravenously injected with fluorescein sodium salt (FITC-Na^+^), a 376Da molecular tracer normally excluded from an intact BBB. BBB permeability was evaluated by measuring the amount of FITC-Na^+^ in the supernatants of homogenized brains (Fig 5A). In *Mal*+/+ mice, ETX treatment significantly increased the amount of FITC-Na^+^ in brain homogenates over two-fold compared to control-treated mice. In comparison, ETX-induced BBB permeability was not observed in *Mal*-/- mice.

To determine the anatomical locations of ETX-induced BBB permeability, control and ETX-treated mice were intravenously injected with FITC-conjugated 70kDA dextran, and permeability determined by microscopic evaluation of cryosections (Fig 5B). Consistent with the FITC-Na^+^ results, only ETX-treated *Mal*+/+ mice experienced BBB permeability. Composite micrographs reveal that *Mal*+/+ ETX-treated mice had significantly higher amounts of 70kDA dextran fluorescence in the brain parenchyma compared to control treated mice and ETX-treated *Mal*-/- mice. In addition, 70kDA dextran can be observed accumulating in the pericallosal white mater and cerebellum of ETX-treated *Mal*+/+ mice. Interestingly, BBB permeably was observed around capillaries, venuels, and veins. In comparison, 70kDA dextran was confined to the blood vessels of control-treated mice and ETX-treated *Mal*-/- mice. When the pericallosal white matter of ETX-treated *Mal*+/+ mice are observed at higher magnification, 70kDa dextran can be observed accumulating in ovoid shaped lesion, perpendicular to the corpus callosum (Fig 5C). Interestingly, this accumulation of 70kDA dextran appears to surround a central vein, depicted as a shadow in the fluorescent dextran halo.

To further evaluate ETX induced BBB permeability, control and ETX treated *Mal*+/+ mice were evaluated for extravasation of endogenous mouse IgG (155kDa) by fluorescent microscopy (Fig 5D). In these experiments, at designated time points following ETX treatment, mice were perfused prior to sacrifice to remove circulating IgG. Extensive IgG extravasation, binding, or internalization on blood vessels were observed in ETX-treated mice, but not saline-treated mice. IgG extravasation was most notable in the pericallosal white matter, especially around larger blood vessels that run perpendicular to the corpus callosum, believed to be deep medullary veins.

Finally, organs from saline and ETX treated *Mal+/+* mice were also evaluated by Haemotoxylin and Eosin (H&E) staining (SF 1). Interestingly, light microscopic examination of brain, heart, lung, liver, kidney, spleen, and intestine did not reveal any significant differences between the two treatment groups. This is consistent with previous published results that found, although mice displayed symptoms of ETX intoxication, no gross or microscopic brain pathology was observed[20]. In comparison, other publications have observed mild to severe renal pathologies including tubular necrosis, pyknotic nuclei, and desquamated epithelia, however these results appear to be both dose and time dependent[35, 37, 45, 55]. This indicates that H&E staining might not be sensitive enough to measure ETX-induced BBB permeability in mice at this acute dose and time point. Taken together, this data indicates that MAL expression is necessary for ETX-induced BBB permeability in mice.

### CAV1 is required for ETX induced BBB permeability

**Fig 6:**
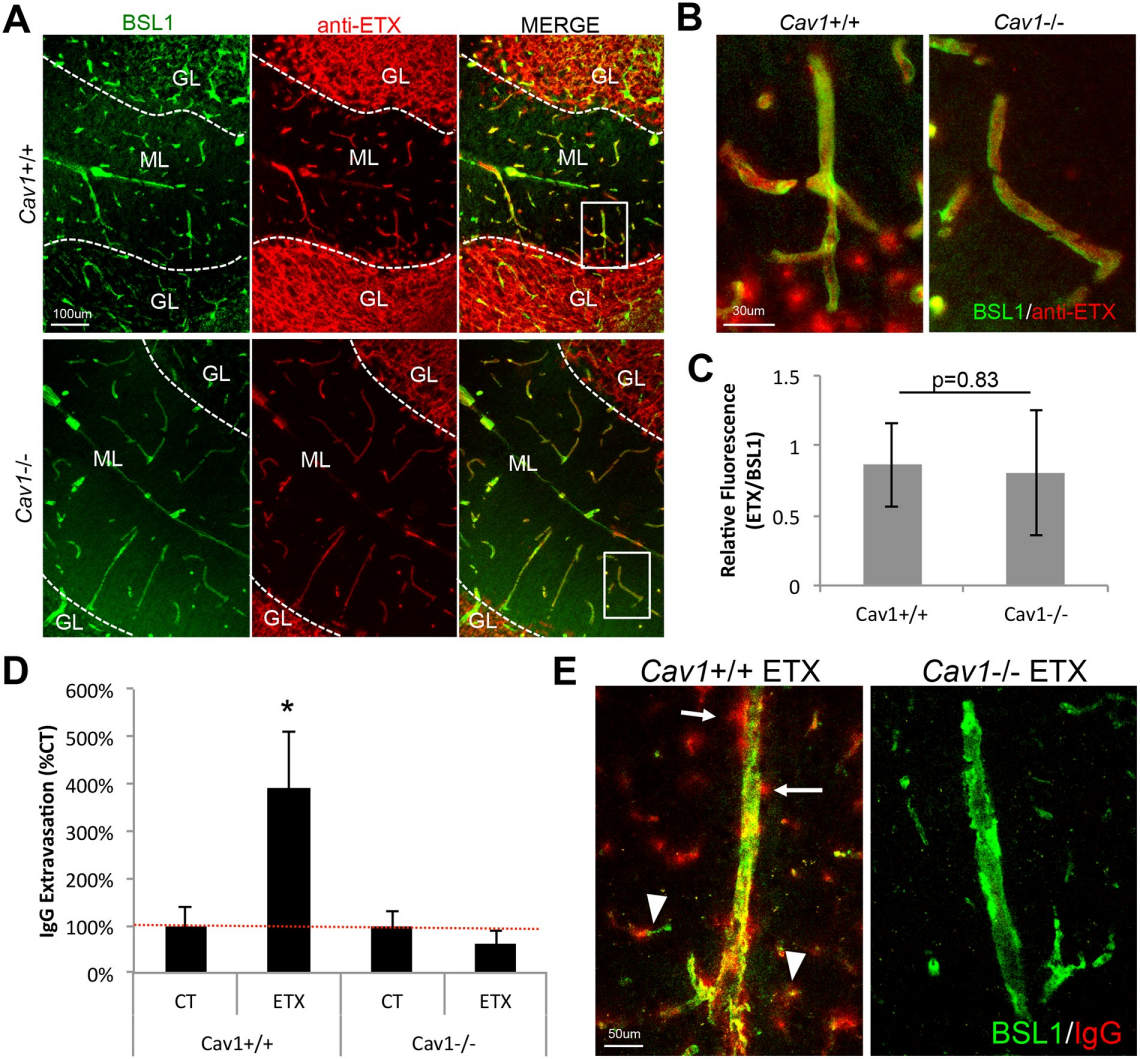
CAV1 expression is necessary for ETX induced BBB permeability but not ETX binding to brain microvasculature. (A) To determine if CAV1 was necessary for ETX binding to brain microvasculature, cerebellum cryosections from *Cav1+/+* and *Cav1-/-* were probed with epsilon protoxin (proETX). ProETX binding was detected by and anti-ETX antibody (red). FITC-BSL1 was used to identify vasculature (green). Note that proETX is observed binding to the microvasculature in the molecular layer (ML) as well as myelin in the granular layer (GL) in both *Cav1*+/+ and *Cav1*-/- mice. (B) Higher magnification images of areas in white boxes in image A. (C) Quantification of proETX binding to vasculature in cerebellum gray matter in *Cav1*+/+ and *Cav1*-/- mice. proETX fluorescence was normalized to BSL1 fluorescence. Results expressed as Mean ± STDEV, *p value determined by T-Test, n = 4–7. D) To evaluate CAV1’s role in ETX induced BBB permeability, *Cav1*+/+ *Cav1*-/- were treated with or without 5ng of ETX per gram of body weight for up to 180 minutes. Mice were perfused with PBS, brains harvested, and cryosectioned. BBB permeability was accessed by immunohistochemistry staining for endogenous mouse IgG in the pericollosal white matter of sagittal sections. Results are normalized to individual genotype controls and expressed as IgG Extravasation (% CT). Results expressed as Mean ± STDEV, * p < 0.05 determined by ANOVA, n = 2–3 mice per group. (E) Representative images from ETX treated *Cav1*+/+ and *Cav1*-/- treated mice. Note in ETX treated *Cav1*+/+ mice, endogenous IgG (red) can be observed leaking for large medullary veins (white arrows) and smaller capillaries (white arrow heads). FITC-BSL1 (green) was used to identify vasculature.

Because CAV1 expression is enriched in endothelial cells and CAV1 has been suggested to play an important role in ETX oligomerization and pore formation[73], we sought to determine CAV1’s role in ETX binding to CNS microvasculature and ETX induced BBB permeability. To determine if CAV1 is necessary for ETX binding, brain cryosections from mice expressing CAV1 (*Cav1*+/+) and mice deficient in CAV1 (*Cav1*-/-) were probed with proETX and proETX binding determined by an anti-ETX antibody (Fig 6A and 6B). It is important to note that proETX and active ETX bind to targets similarly [39, 52]. As expected, proETX was observed to bind to CNS microvasculature and myelinated areas in both *Cav1*+/+ and *Cav1*-/- mice. proETX binding to BEC were evaluated in cerebellar gray matter to reduce potential interference from proETX binding to myelin. Fluorescent quantification of proETX binding to gray matter endothelial cells revealed no difference between *Cav1*+/+ and *Cav1*-/- mice (Fig 6C). In addition, no difference in proETX binding to myelinated structures in *Cav1*+/+ versus *Cav1*-/- mice were observed, although this was not extensively studied. This data confirms results that CAV1 expression is not necessary for ETX binding in mice.

We next sought to determine if CAV1 played a role in ETX-induced BBB permeability. To evaluate BBB permeability in treated *Cav1*+/+ and *Cav1*-/- mice, endogenous IgG extravasation in pericallosal white matter after perfusion was evaluated by fluorescent microscopy in cryosections (Fig 6D). *Cav1*+/+ mice treated with ETX had almost four times more IgG extravasation compared to *Cav1*+/+ control-treated mice. In comparison, no increase in IgG extravasation was observed in ETX-treated *Cav1*-/- mice compared to control-treated *Cav1*-/- mice. In addition, fluorescent microscopy revealed a propensity to cause BBB permeability in deep medullary veins in ETX-treated *Cav1*+/+ mice (Fig 6E). IgG extravasation can also be observed outside larger vessels, as well as forming halos around smaller vessels. This data indicates that CAV1 is necessary for ETX-induced BBB permeability but not binding in mice.

### ETX-induced BBB permeability involves caveolae-dependent transcytosis

**Fig 7:**
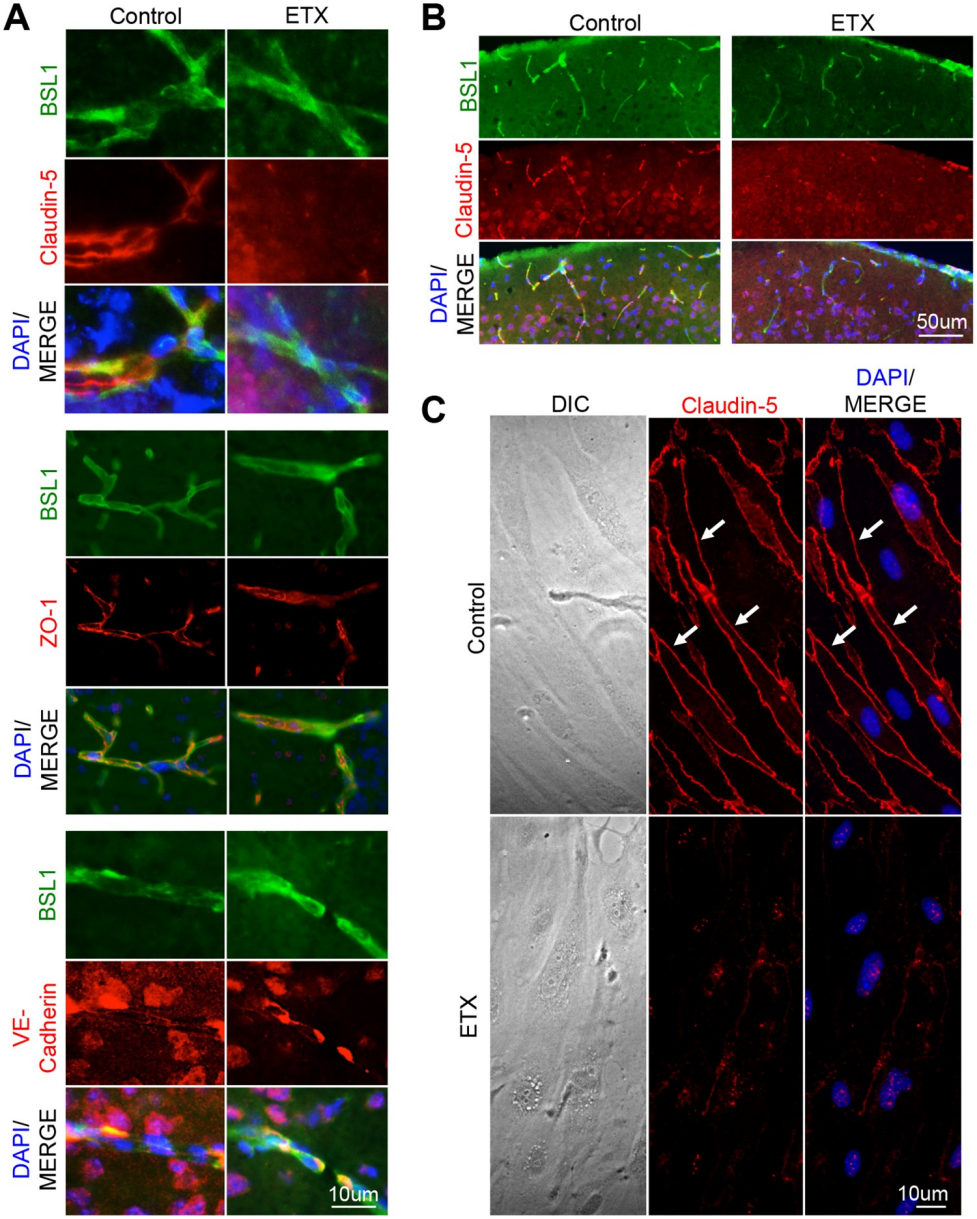
ETX treatment causes a down regulation in claudin-5 staining. Mice were treated with 5ng of ETX per gram body weight for one hour and then perfused with PBS. Saline treated mice were used as controls. (A) Cyrsosections were evaluated for tight junction markers including claudin-5, ZO-1, and VE-cadherin. FITC-BSL1 (green) was used to visualize vasculature. (B) Extensive reduction in claudin-5 staining was also observed in ETX treated animals compared to controls. Sagittal sections of cortical matter. (C) Down regulation of claudin-5 was also confirmed *in-vitro*. Primary mouse BEC were treated with or without 50nM ETX for two hours and then stained for claudin-5. Under control conditions, claudin-5 is found at cell-cell junctions (white arrows). When treated with ETX, claudin-5 treatment significantly decreases and becomes perinuclear.

It is important to note that ETX opens the BBB to a wide range of molecular tracers including 376Da FITC-Na, 70kDA dextran, and 155kDA endogenous IgG (Figs 5 and 6). To determine the mechanisms involved in opening the BBB to such a wide range of different sized molecules, we first evaluated if ETX-treatment influenced paracellular permeability at the BBB. As TJ play an important role inhibiting paracellular permeability in BEC, we evaluated TJ and associated protein staining in brains of wild type (WT) mice treated with or without ETX (Fig 7A). ZO-1 and VE-cadherin staining was similar in control- and ETX-treated mice. However, ETX-treated mice demonstrated a marked decrease in claudin-5 staining. In addition, down regulation of claudin-5 was observed throughout the cortex of ETX-treated mice (Fig 7B), and was confirmed *in vitro* using primary murine BEC (Fig 7C). This data suggests that ETX may cause BBB dysfunction by affecting paracellular permeability. However, selective loss of claudin-5 expression in mice opens the BBB to molecules smaller than 800 Daltons (D) [83]. In comparison, ETX opens the BBB to molecules much larger than 800D, including 70kD dextran and 155kDA IgG. Therefore, the loss of only claudin-5 and its specific paracellular function does not explain how ETX is able to induce barrier opening to such a wide range of different sized molecules, suggesting transcellular pathways are involved in ETX induced BBB permeability. In other words, ETX may cause BBB permeability by increasing macropinocytosis, clathrin-mediated transcytosis, or caveolae-mediated transcytosis. Interestingly, down regulation of claudin-5 via CAV1 and/or caveolae has been reported under certain pathological and experimental conditions[84–87].

**Fig 8:**
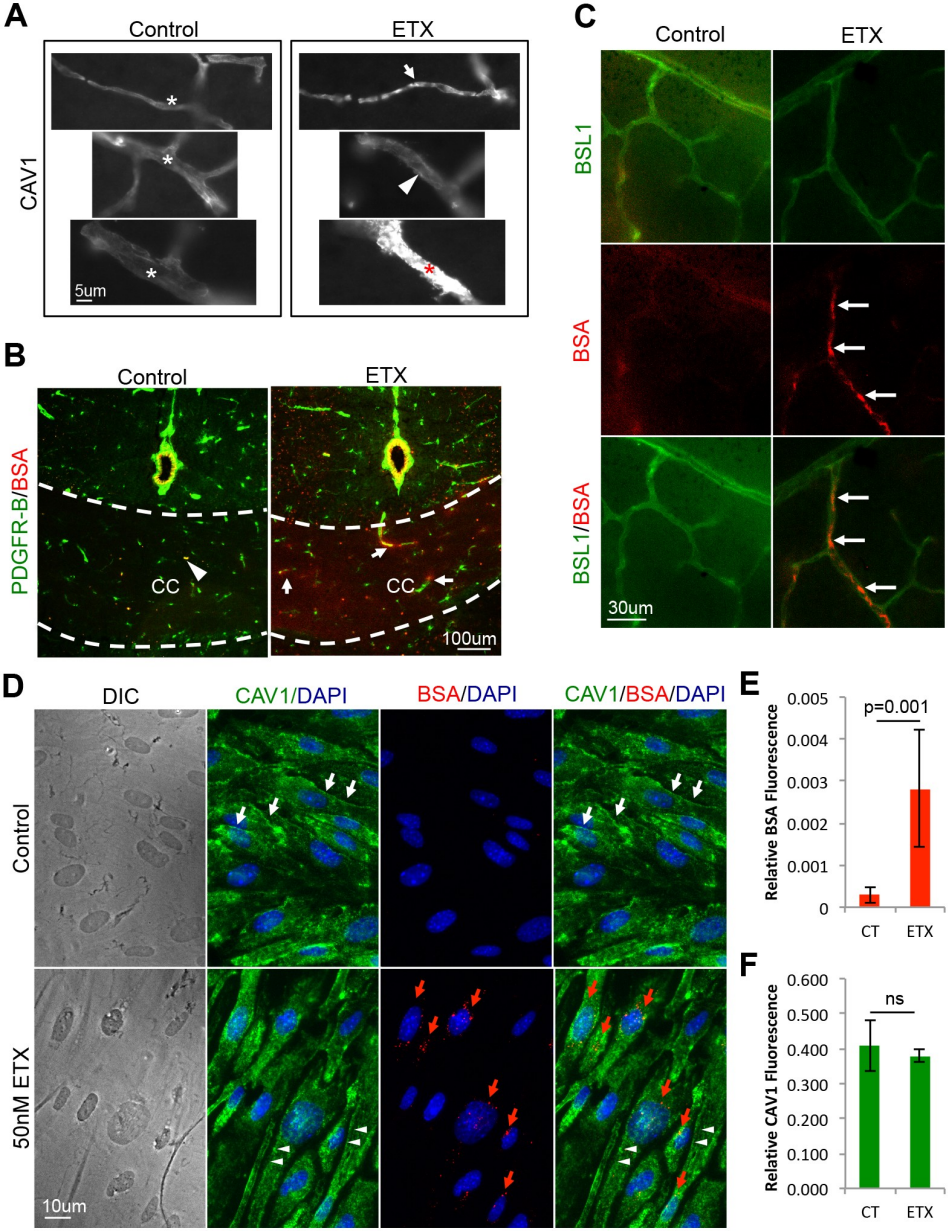
ETX treatment causes an increase in caveolae dependent BBB permeability and internalization. (A) Wild type mice were treated with 5ng ETX per gram of body weight for 1 hour. CAV1 staining was evaluated by IHC. White asterisks denote normal CAV1 staining in various sized blood vessels. In ETX treated mice, aberrant CAV1 staining was observed in some small capillaries as punctate staining (white arrow). In some medium sized vessels, CAV1 staining appeared basally located (white arrow head). In some large vessels, a dramatic increase in CAV1 staining was observed (red asterisk). (B) After ETX treatment, mice were intravenously injected with BSA-594 and then perfused. Coronal sections in control treated mice revealed BSA-594 (red) confined to vasculature lumen (white arrow head). In ETX treated mice, BSA-594 extravasation was observed accumulating in the corpus collosum (CC, thickness denoted by white dotted line) and as halos surrounding smaller vessels (white arrows). PDGF receptor-beta (green) was used to identify pericytes/vasculature. (C) Retinal whole mounts of control and ETX treated mice reveal increased internalization of BSA-594 by retinal endothelial cells after ETX treatment (white arrows). FITC-BSL1 (green) was used to identify vasculature. (D) Primary BEC were treated with our without 50nM ETX for 2 hours in the presence BSA-594 and CAV1 staining was evaluated by ICC. Internalized BSA-594 was observed in perinuclear organelles in ETX treatment cells (red arrows). In control treated cells, CAV1 staining can be observed extending all the way to cell-to-cell contacts (white arrows). After ETX treatment, CAV1 is absent from cell-to-cell contacts (white arrow heads). (E) and (F) Fluorescent quantification of CAV1 and BSA-594, respectively. Results expressed as Mean ± STDEV, p values determined by T-Test, n = 4.

Because ETX-induced BBB permeability requires expression of CAV1, a key component in caveolae formation [88, 89], we sought to determine if ETX causes an increase in BBB permeability via caveolae dependent-transcytosis of albumin[90–92]. First, CAV1 expression in brain vasculature was evaluated after ETX-treatment (Fig 8A). In control treated mice, CAV1 staining in brain microvasculature is smoothly distributed throughout vessels of various sizes. In ETX-treated mice, CAV1 staining is disrupted. In some small vessels, CAV1 staining becomes punctate and increases in intensity. Alternatively, in medium vessels, CAV1 appears to localize to the basal surface of endothelial cells. When mice were intravenously injected with Alexaflour-594 fluorescently labeled bovine serum albumin (BSA-594) after control- or ETX-treatment, an increase in BSA-594 extravasation is observed in ETX-treated mice compared to controls (Fig 8B). In control-treated mice, BSA-594 was confined to the vasculature lumen. In addition, increased BSA-594 internalization by CNS vasculature was observed in the retina of ETX-treated mice compared to controls (Fig 8C). This data confirms previous results that ETX causes an increase in albumin extravasation *in vivo* [46, 49, 51, 53, 66, 67].

These results were confirmed *in vitro* using primary murine BEC (Fig 8D and 8E). BEC were treated with or without 50nM of ETX for two hours in the presence of BSA-594 and internalization was measured by immunofluorescence intensity (Fig 8E). Under control conditions, very little BSA was internalized (Fig 8D). However, ETX-treatment caused a significant increase in BSA-594 internalization and could be found accumulating in perinuclear vesicles (Fig 8D). No difference in CAV1 staining intensity was observed (Fig 8F), however ETX-treatment did change the localization of CAV1 (Fig 8D). In control-treated cells, CAV1 staining is observed throughout the cell, with a slight accumulation at cell junctions. In ETX treated cells, CAV1 staining has retracted from cell-to-cell contacts. Taken together with the CAV1-/- *in vivo* results, this data indicates that ETX causes an increase in albumin internalization in murine BEC.

**Fig 9:**
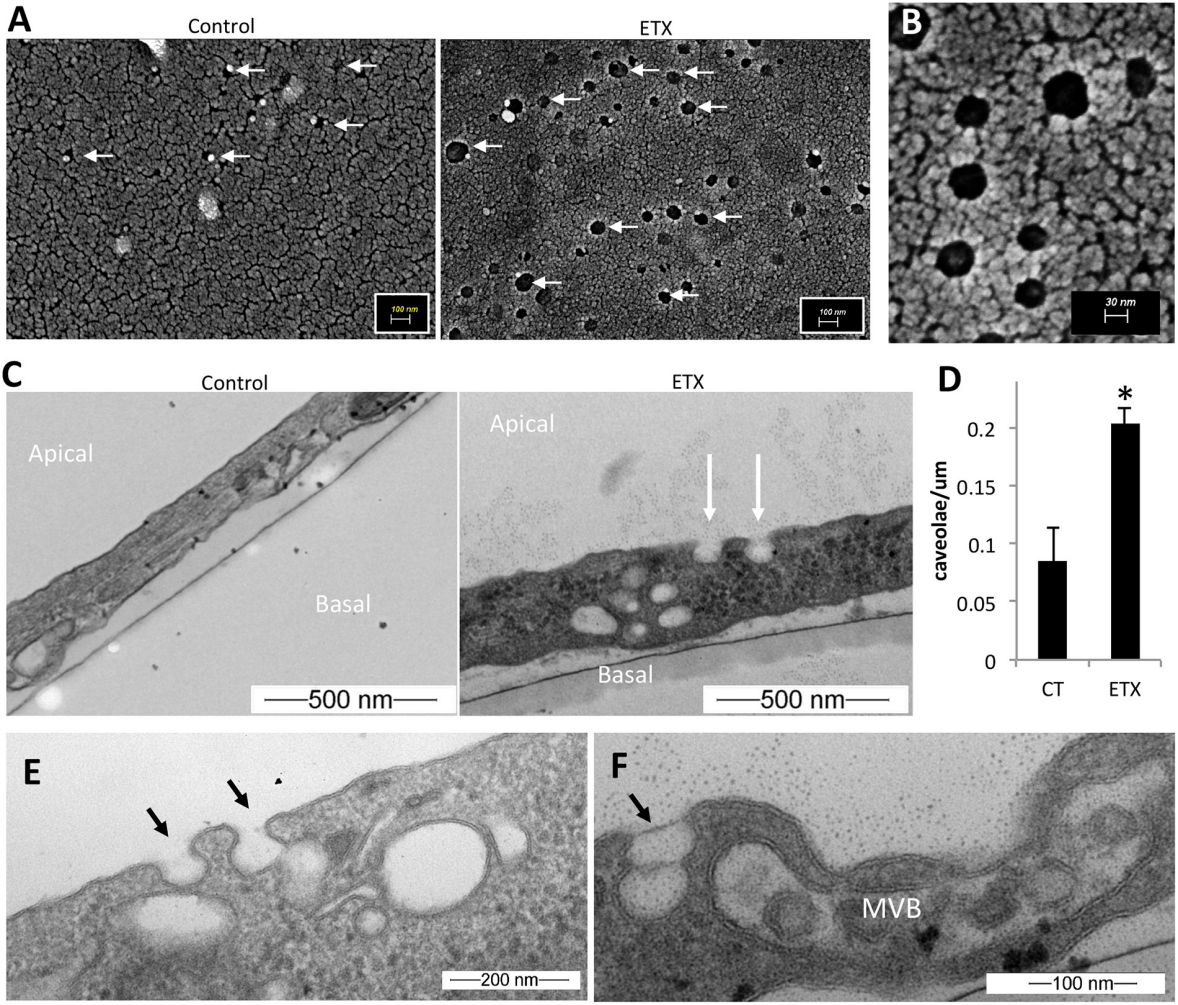
ETX treatment causes caveolae formation. (A) SEM of primary BEC treated with or without 50nM of ETX for 1 hour. White arrows point to apical surface invaginations. (B) Higher magnification of surface invaginations on ETX treated BEC. (C) Vertical TEM sections of primary BEC treated with or without 50nM ETX for 2 hours. White arrows point to apical surface invaginations morphologically similar to caveolae. (D) Quantification of the number of caveolae per um of cell surface in control or ETX treated cells. Results expressed as Mean ± STDEV, *p<0.003 determined by T-Test, n = 3. (E) and (F) additional micrographs of BEC treated with 50nM ETX for 2 hours. Black arrows denote caveolae fusing into other internalized caveolae or endosomes. In some cells, large MVB are observed.

To determine if ETX treatment induces caveolae formation, control and ETX treated BEC were examined for the presence of caveolae using electron microscopy. In control BEC, SEM revealed occasional invaginations approximately 40–50nm wide (Fig 9A). In comparison, ETX-treated cells had numerous invaginations ranging in diameter from approximately 40–100nm in size (Fig 9A and 9B). Vertical TEM sections displaying both the apical and basal BEC membranes revealed limited apical invaginations in control-treated cells (Fig 9C). In comparison, ETX-treated cells demonstrated numerous apical invaginations. These invaginations, with a flask-shaped morphology and non-electron dense membrane, are characteristic of caveolae[88, 92–96]. ETX-treatment significantly increased the number of caveolae on the apical cell surface compared to control treated cells (Fig 9D). Interestingly, many caveolae observed in ETX-treated cells could be seen fusing with other internalized caveolae, often near larger endosomes and multivesicular bodies (MVB) (Fig 9E and 9F). This data indicates that ETX induces caveolae formation in murine BEC.

### ETX causes MAL and Caveolae-dependent vesicle formation in BEC without cell death

**Fig 10:**
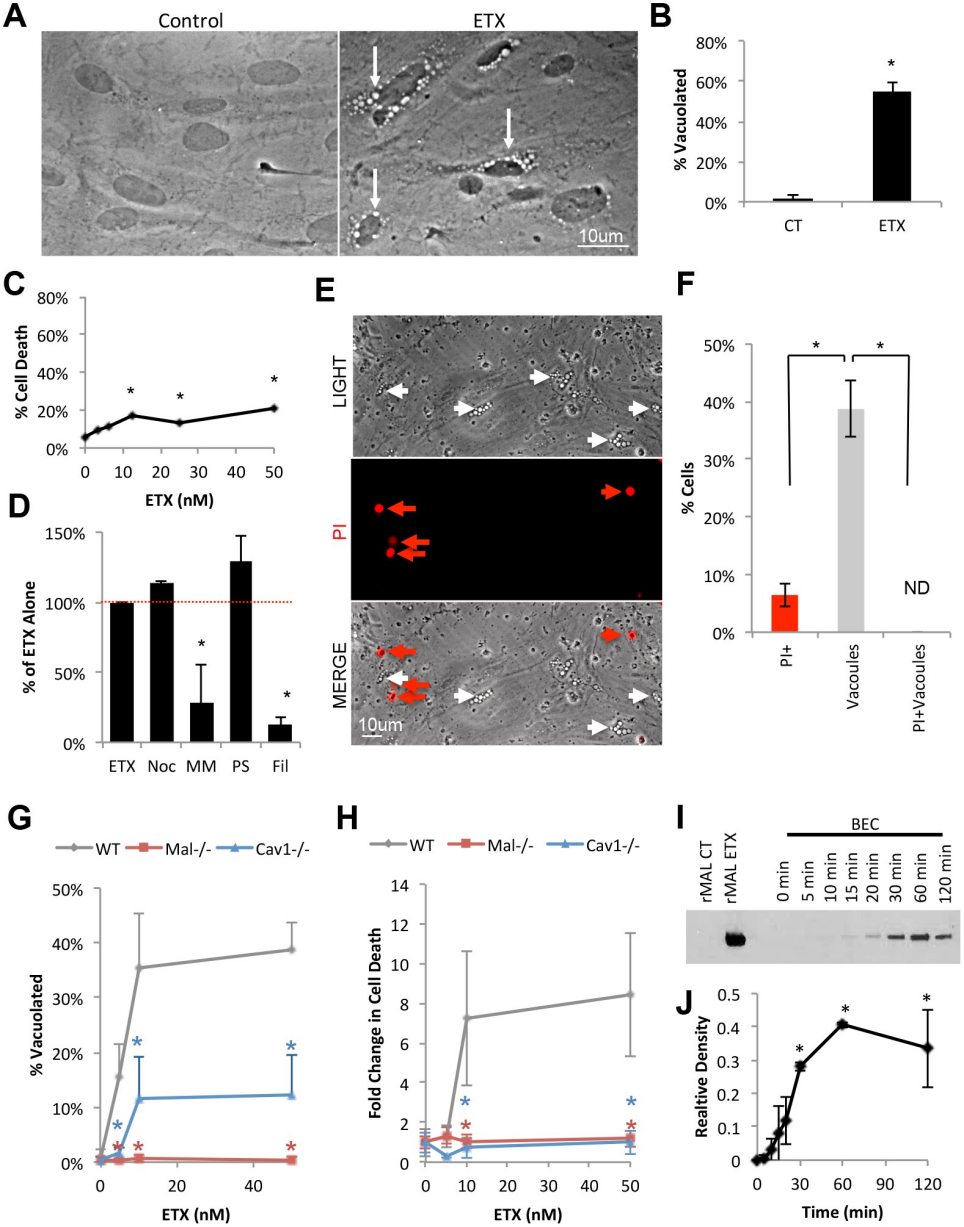
ETX treatment causes vacuolation in primary BEC without cell death. (A) *In vitro* ETX treatment of primary BEC revealed large, perinuclear vacuole formation (white arrows) compared to untreated controls. (B) Quantification of the number of vacuolated cells in control or ETX treated BEC after two hours. Results expressed as Mean ± STDEV, *p<0.001 determined by T-Test, n = 4. (C) Cell death was evaluated in BEC after 4 hours of ETX treatment at indicated doses. Cells were treated, trypsinized, and evaluated for cell death via PI inclusion by flow cytometry. Results are expressed as the number of PI positive cells by the total number of cells (% cell death). Results expressed as Mean ± STDEV, *p<0.05 versus untreated control, determined by ANOVA, n = 3. (D) To determine if macropinocytosis, dynamin, clathrin-coated pits, or caveolae are necessary for ETX induced vacuolation, BEC were pretreated with nocadozale (Noc), Pitstop (PS), MiTMAB (MM), or filipin (Fil), respectively, for 30 minutes prior to ETX treatment. Results are expressed as the number of vacuolated cells compared to the number of vacuolated cells when treated with ETX alone (% of ETX Alone). Results expressed as mean ± STDEV of at least two separate experiments performed in triplicate. *p<0.01 versus untreated control, determined by ANOVA. (E) Live imaging of BEC cells treated with 50nM of ETX for 4 hours. PI was used to identify dead cells (red arrows). Note that vacuolated cells (white arrows) are not PI positive. (F) Quantification of the percent of cells that are PI positive only (PI+), contain vacuoles only (vacuoles), or both (PI+Vacuoles). Results expressed as mean ± STDEV, *p<0.01 determined by ANOVA, n = 5–6. (G) and (H) BEC were isolated from WT, *Mal*-/-, or *Cav1*-/- mice, and incubated with indicated ETX doses for 4 hours, and cell vacuolation or cell death were evaluated by live cell imaging, respectively. Cell death was expressed as the number of PI positive cells compared to untreated controls (% CT). Results are the mean ± STDEV. *p<0.01 determined by ANOVA versus WT, n = 5–6. (I) Evaluation of ETX oligomerization in primary BEC via western blot. BEC were treated with 50nM of ETX for indicated time points and whole cell lysates were evaluated. CHO cells expressing MAL (rMAL) treated with or without 25nM ETX for 30 minutes were used as positive and negative controls, respectively. ETX oligomers are approximately 150kDa. (J) Densitometry readings of ETX oligomers in BEC normalized to ETX oligomer detected in ETX treated CHO cells. Results are the mean ± STDEV. *p<0.05 determined by ANOVA versus 0 min, n = 2.

ETX treatment was also observed to cause perinuclear vacuolation in BEC compared to control-treated cells (Fig 10A). After two hours of 50nM ETX treatment, over 55% of ETX treated BEC were observed to be vacuolated compared to 1% for control-treated BEC (Fig 10B). Surprisingly, only a limited amount of cell death was observed when cells were evaluated by PI inclusion assay via flow cytometry after four hours of ETX treatment (Fig 10C). To determine if BEC vacuolation was dependent on caveolae formation, BEC were pretreated with pharmacological inhibitors of macropinocytosis (nocandozole, a tubulin-binding agent that disrupts microtubule assembly and disassembly), clathrin-coated pits (PitStop, a clathrin-specific inhibitor), and caveolae (filipin, a cholesterol sequestration agent that interferes with lipid raft structure) (Fig 10D). Cells were also treated with the dynamin inhibitor, MiTMAB, which is necessary for both clathrin-mediated and caveolae-mediated transcytosis. Only pretreatment with filipin and MiTMAB inhibited ETX-induced vacuolation, indicating that lipid rafts/caveolae and dynamin are both necessary for this process. Finally, cell death in vacuolated cells was also evaluated. Live imaging analysis of ETX-treated BEC revealed that vacuolated cells are not PI positive, indicating that vacuolation does not result in BEC death and moreover, that vacuolation was associated with viability (Fig 10E and 10F).

The role of MAL and CAV1 in ETX-induced vacuolation and cell death in BEC were also evaluated. BEC isolated from WT, *Mal*-/-, and *Cav1*-/- mice were treated with indicated doses of ETX for four hours and evaluated for vacuolation and cell death via PI inclusion. When treated with 50nM of ETX, 39% of WT BEC were vacuolated, while only 12% and 0.3% of *Cav1*-/- and *Mal*-/- cells were vacuolated, respectively (Fig 10G). In addition, WT BEC treated with 50nM of ETX for four hours experienced an eight-fold increase in PI+ cells compared to control BEC (Fig 10H). In comparison, there was no significant increase in the amount of cell death in ETX-treated *Cav1*-/- or *Mal*-/- BEC compared to control cells.

Finally, as ETX oligomerization/pore formation has been postulated as being required for ETX-induced cytotoxicity, ETX-oligomerization in whole cell lysates from BEC was confirmed via western blot (Fig 10I and 10J). Whole cell lysates from CHO cells expressing MAL were used as positive controls for ETX oligomerization [97]. ETX oligomers of approximately 150 kDA were observed in BEC and appeared to be time-dependent. ETX oligomerization in BEC appeared to peak 60 minutes after ETX treatment.

Using murine BEC, this data indicates that ETX causes 1) BEC vacuolation via a caveolae-dependent mechanism without causing cell death, 2) MAL and CAV1 are required for both ETX-induced vacuolation and cell death, and 3) ETX forms oligomers in BEC.

### ETX-induced vacuoles are late endosomes and multivesicular bodies (MVB)

**Fig 11:**
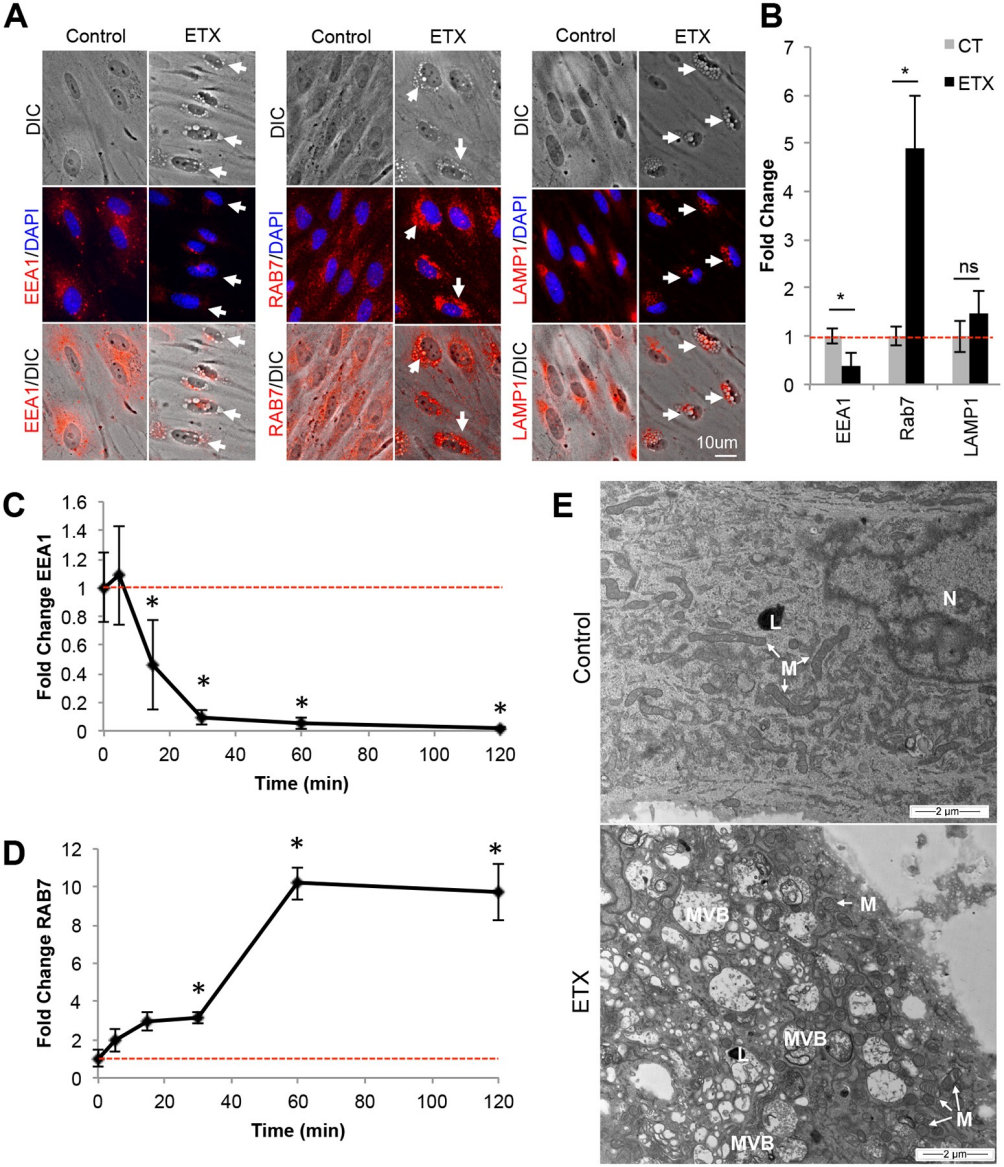
ETX treatment causes formation of RAB7+ endosomes and MVB. (A) Primary BEC were treated with 50nM ETX for 2 hours. Cells were stained with anti-EEA1, RAB7, or LAMP1. White arrows point to vacuolated BEC. (B) Fluorescence intensity analysis of EEA1, RAB7, and LAMP1 with and without ETX treatment. Fluorescence was normalized to untreated controls and expressed as Fold Change. Results are the mean ± STDEV. *p<0.05 determined by T-Test, n = 3–6. Changes in EEA1 (C) and RAB7 (D) staining intensity was also evaluated at indicated timepoints after 50nM ETX treatment. Fluorescence was normalized to untreated controls (0 min) and expressed as Fold Change. Results are the mean ± STDEV. *p≤0.05 determined by ANOVA versus 0 min, n = 3–6 (E) Horizontal TEM sections near the basal side in control or ETX BEC reveal ETX induced formation of numerous MVB. No increase in lysome (L) numbers was observed. Mitochondria (M) in ETX treated cells appear rounded and swollen compared to the elongated mitochondria in control treated cells.

To determine the nature of ETX-induced vacuoles in BEC, BEC were evaluated for the presence of different endosome markers via ICC. Cells were stained with various endosome markers including EEA1 for early endosomes, RAB7 for late endosomes, and LAMP1 for lysosomes (Fig 11A and 11B). 50nM ETX treatment significantly reduced EEA1 staining intensity while significantly increasing RAB7 staining after two-hour treatment compared to control cells. Importantly, ETX induced vacuoles were positive for RAB7. LAMP1 staining intensity was not affected, however some ETX induced vacuoles did stain positive for LAMP1 around the edges. To confirm the effect ETX on the lysosome population, live staining of BEC were performed using a fluorogenic probe that accumulates in acidic organelles (SF 2A). No difference in staining intensity was observed between control- and ETX-treated cells, indicating that ETX treatment does not increase lysosome activity at the time points tested (SF 2B). In addition, RAB5, a marker for early and/or sorting endosomes, and RAB11, a marker for recycling endosomes, were also evaluated via ICC (SF2C and D, respectively). No difference in RAB5+ or RAB11+ endosome localization or intensity was observed in control- versus ETX-treated BEC.

To further elucidate changes in endosome populations, a time course examining changes in EEA1+ and RAB7+ staining was performed (Fig 11C and 11D, respectively). A significant decrease in EEA1 staining was observed 15, 30, 60 and 120 minutes after ETX treatment (Fig 11C). In comparison, a significant increase in RAB7 staining was observed 30, 60 and 120 minutes post ETX treatment. This data indicates that ETX causes changes in the EEA1+ endosome population prior to the RAB7+ endosome population. Interestingly, these results correspond with ETX oligomerization in ETX treated BEC (Fig 10I and 10J).

To further characterize ETX-induced vacuoles in BEC, control- and toxin- treated BEC were evaluated by TEM. Horizontal TEM sections near the basal surface of BEC revealed numerous MVB in ETX-treated BEC (Fig 11E). Because of their perinuclear location and electron-sparse nature, MBV observed via TEM are believed to be the phase-bright vacuoles observed in ETX-treated BEC (Fig 10A). Since BEC are grown on glass coverslips instead of their physiological, semi-permeable, parenchymal environment, we believe that this large accumulation of MVB near the basal surface is a form of “frustrated transcytosis”, in which the impenetrable glass barrier is inhibiting exocytosis of vesicles at the basal surface. Consistent with previous results, there was no observed increase in lysosome numbers or size after ETX treatment. However, mitochondrial abnormalities were observed in ETX-treated BEC compared to control-treated cells, confirming previous results [48, 71, 98]. Taken together, this data indicates that ETX causes a decrease in EEA1+ early endosomes and increases RAB7+ late endosomes and MVB in murine BEC.

## Discussion

**Fig 12:**
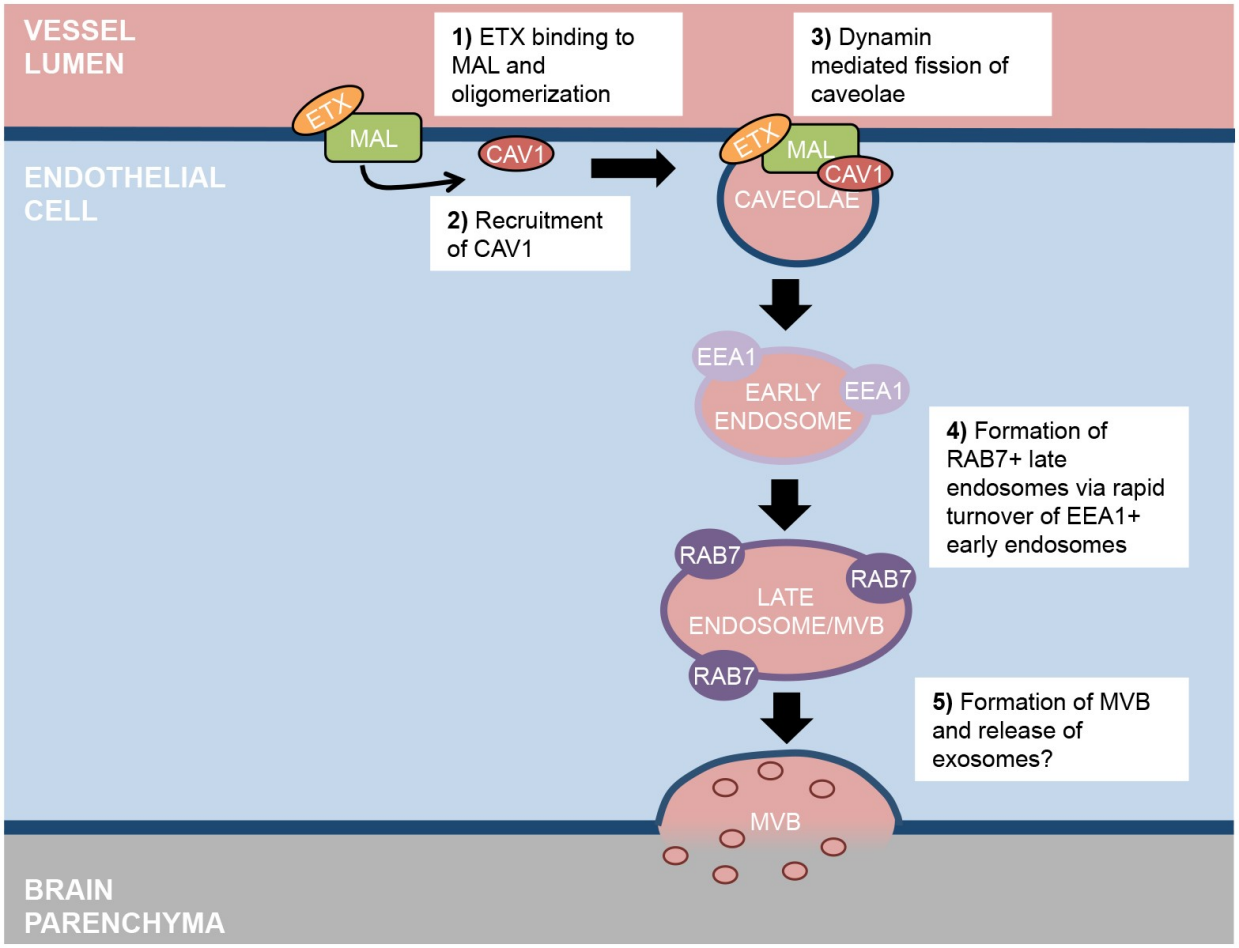
Proposed mechanism of ETX induced transcytosis. (1) ETX binds to MAL and oligomerizes, (2) causing recruitment of CAV1. (3) Recruitment of CAV1 results in increased caveolae formation and internalization and is dependent on dynamin. (4) Internalized caveolae, containing various blood-borne material, rapidly fuse with EEA1+ early endosomes, which traffic contents to RAB7+ late endosomes, (5) ultimately forming MVB. We propose that MVB then fuse with the basal membrane of the endothelial cells, releasing their contents into the brain parenchyma.

In this study, we have identified a cellular mechanism involved in ETX-induced BBB permeability in mice. We demonstrate that ETX selectively binds to the microvsacualture of the CNS, and this binding is dependent on expression of the myelin and lymphocyte protein MAL. In addition, MAL expression is required for ETX induced BBB permeability *in vivo*. We also observed that ETX opens the BBB to molecular tracers ranging from less than 400Da up to 155kDa. ETX-induced BBB permeability does not appear to be a result of overall BEC cell death, as very limited cell death was observed in ETX treated BEC *in vitro*. However, ETX induced BEC death may still play a role in ETX induced BBB permeability *in vivo*, as death of even a small percentage of BEC may significantly impact the structure of the BBB. In addition, ETX-mediated BBB permeability does not appear to be regulated by an increase in paracellular permeability. Although ETX-treatment specifically reduced claudin-5 expression, ETX-induced BBB permeability was not mediated by increased paracellular flux, as loss of claudin-5 only opens the BBB to molecules smaller than 800 Da. Instead, ETX-induced BBB permeability appears to be a result of increased transcytotic activity, as examination of primary BEC revealed that ETX treatment caused an increase in caveolae formation and internalization. This was supported by the observation that CAV1, an important component of caveolae, was necessary for ETX induced BBB permeability *in vivo*, but not binding. ETX treatment resulted in a rapid decrease of EEA1+ early endosomes followed by an accumulation of large RAB7+ late endosomes and MVB. Taken together, we believe that ETX causes BBB permeability by binding to the apical side of CNS microvasculature via interactions with MAL (Fig 12). ETX oligomerization causes recruitment of CAV1, resulting in increased caveolae formation and internalization. Internalized caveolae, containing various blood-borne material, rapidly fuse with early endosomes, which traffic contents to late endosomes and MVB. We propose that MVB then fuse with the basal membrane of the endothelial cells, releasing their contents into the brain parenchyma.

Although there are numerous different types of endocytic and transcytotic pathways, only three have been definitively confirmed in BEC including macropinocytsis, clathrin-coated pits, and caveolae [13]. Under normal conditions, all three pathways are severely diminished in BEC compared to peripheral endothelial cells; however, inflammatory and pathogenic stimuli have been shown to increase vesicle formation in BEC. Transcytosis is a complex process, and these pathways can often overlap. In terms of ETX-induced transcytosis, the process appears to be receptor-mediated and dependent on both lipid rafts and dynamin, as pharmacological inhibition of lipid rafts and dynamim both prevented ETX-induced vacuolation of BEC. This is consistent with caveolae mediated transcytosis, which requires both lipid rafts and dynamin [90, 93, 99]. These results, when considered with the observation that mice deficient in CAV1 are resistant to ETX-induced permeability, strongly indicate that ETX induces caveolae-mediated transcytosis in CNS microvasculature. Ultrastructure analysis confirmed increased caveolae formation in ETX-treated BEC compared to untreated controls. Interestingly, an increase in pinocytic vesicles in brain endothelium has been briefly mentioned before in ETX-treated mice[48]. Although caveolae formation is mediated by ETX binding, we believe ETX-induced caveolae contain other blood-borne material via adsorptive-mediated transcytosis and/or fluid-phase transcytosis [14], explaining why ETX causes extravasation of so many different sized blood-borne materials.

ICC analysis revealed a rapid decline in EEA1+ endosomes and accumulation of large, RAB7+ late endosomes, viewed as large, phase-bright vacuoles via light microscopy. TEM identified these vacuoles as large, basally oriented MVB. Interestingly, similar results have also been observed in polarized MDCK cells[100]. In that study, authors observed ETX-mediated vacuolation of MDCK cells and discovered that internalized ETX colocalizes with EEA1+ positive endosomes as well as RAB7+ endosomes. Contrary to our results however, was the observation that ETX treatment increased lysosome concentration and acidic activity in MDCK cells. Finally, accumulation of RAB7+ endosomes and vacuoles has also been observed in CHO cells expressing MAL as well [97]. This indicates that although different cell types may share some ETX-induced endocytic pathways, the ultimate fate of these endosomes may be cell specific.

By using mice that are deficient in MAL, we demonstrate that MAL is necessary for both ETX binding to CNS microvasculature and ETX-induced BBB permeability. This data adds to the growing body of literature that strongly suggests MAL is the putative ETX receptor. By using ETX as a MAL specific ligand, we observe that MAL expression seems to be limited to CNS microvasculature, and to a lesser extent, intestinal microvasculature. The observation that MAL is exclusively expressed in these endothelial cells helps illuminate two poorly understood questions about ETX-mediated disease; the first being how ETX moves from the intestinal lumen into the bloodstream and the second being why ETX preferentially accumulates in brains of afflicted animals. Although ETX absorption into the bloodstream from the intestinal lumen is a well-accepted process, how this occurs without causing overt intestinal damage in mice has not been determined [32, 34]. MAL expression in murine intestinal endothelial cells may help explain how toxin enters the bloodstream from the intestinal lumen after passing through the intestinal epithelial, possibly using similar endocytic mechanisms as those observed in BEC. The accumulation of ETX in brains of intoxicated mice is most likely due to increased expression of the ETX receptor, MAL, in CNS microvasculature. Expression of MAL in CNS microvasculature allows preferentially binding of ETX in the brain and subsequent accumulation in the brain parenchyma after inducing BBB permeability. The exact role of MAL in endothelial cells is unknown at this time, but it seems reasonable that MAL would play a similar role in apical protein sorting and lipid raft maintenance in BEC as it does in other polarized cell types[101–108].

We also demonstrate that CAV1 is necessary for ETX-induced BBB permeability, but not binding to CNS microvasculature. These results are consistent with an earlier publication that determined CAV1 is not necessary for ETX binding to kidney epithelial cells, but plays an important role in ETX oligomerization[73]. Knockdown of CAV1 in these cells reduced ETX cytotoxicity and oligomerization but did not completely abolish these functions. Importantly, cells naturally deficient in CAV1 have also been shown to be sensitive to ETX cytotoxicity and form ETX oligomers, indicating that CAV1 is not necessary for these processes to occur[75, 109]. It is possible that ETX-sensitive cells that express CAV1 will form caveolae in an attempt to remove ETX pores from the cell membrane as a repair mechanism, as has been observed with other pore-forming toxins[110–113]. Although CAV1 localization is enriched in caveolae, CAV1’s exact function in caveolae formation and internalization is unclear. Recently, CAV1 has been implicated in stabilizing caveolae at the plasma membrane, thereby preventing caveolae internalization. Interestingly, both phosphorylation and dephosphorylation of caveolae components including CAV1 have been implicated in caveolae internalization[88, 92–96, 99, 114, 115]. Because ETX has been demonstrated to cause a rapid decrease in intracellular ATP in various cell types[59, 71, 109], it seems likely that ETX-induced caveolae formation may be regulated by dephosphorylation of caveolae components via indirect or direct mechanisms. The exact role of CAV1 in ETX-mediated caveolae formation as well as its possible interaction with MAL is an area of ongoing research.

In summary, we have identified a cellular mechanism involved in ETX induced BBB permeability in mice. It remains unknown if similar mechanism are involved in ETX-induced BBB permeability in ruminants such as sheep and goats, and the role of MAL and caveolae-mediated transcytosis in these species needs to be evaluated. Nevertheless, these findings help elucidate a processes involved in ETX mediated BBB dysfunction in mice and provide possible therapeutic targets to prevent ETX mediated death of whole animals. Future research is focused on identifying molecular mechanisms involved in ETX-mediated caveolae formation as well as endocytic trafficking.

## Material and methods

### Ethics statement

All animal studies were approved by the Institutional Animal Care and Use Committee of Weill Cornell Medical College, protocol number 2012–0030. These policies and procedures have been developed based on federal regulations and laws mandated and enforced by the Animal Welfare Act, United States Department of Agriculture Animal and Plant Health Inspection Service, The National Institute of Health’s Office of Laboratory Animal Welfare, the Policy of the Public Health Service, American Veterinary Medical Association Guidelines on Euthanasia, The National Institute of Health Revitalization Act, and the United States Government. Ketamine and xylazine were used for anesthesia and euthanasia for terminal in vivo studies. Carbon dioxide inhalation was used for euthanasia to harvest brains for primary cell culture.

### Gene expression analysis

Gene expression in different brain cell types was analyzed by the translating ribosome affinity purification (TRAP) method as previously described [78, 116]. Briefly, transgenic mice expressing EGFP-tagged ribosomal protein L10a (EGFP-L10a) in endothelial cells (Abcb1a-bacTRAP ES3026), pericytes (Gdnf-bacTRAP), astrocytes (Aldh1l1-bacTRAP JD130), oligodendrocytes (Olig2-bacTRAP JD97) L5b pyramidal cells (Colgalt2-bacTRAP DU9), or inhibitory interneurons (Dlx1-bacTRAP GM520) were used to affinity purify cell type specific polysomes from cortex. Each of these BAC transgenic lines has been described in detail previously and transgene expression can be viewed at www.gensat.org/TRAP_listing.jsp, except Gdnf-bacTRAP ES2243 which is described in detail in a forthcoming paper. Polysome-bound mRNAs from endothelial cells, pericytes, L5b pyramidal cells, interneurons, and mRNAs from whole tissue were analyzed by RNA sequencing. Oligodendrocyte TRAP mRNAs and matching whole tissue mRNAs were previously analyzed by microarray using the Affymetrix Mouse Genome 430 2.0 platform [78].

### ETX labeling and activation

Unlabeled proETX was acquired from BEI and activated using immobilized trypsin, TPCK Treated, agarose resin (Thermo Fischer Scientific). 125μL of resin was washed three times in 10mM sodium phosphatase buffer (pH 7.98) then resuspended in 200μL sodium phosphatase buffer. Resuspended resin was combined with 500μL of BEI proETX (0.5 mg/mL) for two hours at 37°C with gentle agitation. The preparation was centrifuged at 18,000 rcf for 10 min and the supernatant containing the activated ETX harvested. Activated toxin (11 μM) was aliquoted and stored at −80°C until use. In other preparations proETX was fluorescently labeled using Alexa Fluor 594 Protein Labeling Kit per the manufactures instructions (ThermoFisher Scientific) and activated using the same method. Fluorescent labeling did not alter ETX cytotoxicity or binding.

### Intravenous injection of fluorescently Labeled ETX

Wild type mice (C57BL/6J, The Jackson Laboratories) or *Mal*-/- mice (generated by replacing the first exon of the mal gene with the lacZ gene sequence by standard embryonic stem cell technology)[82] were deeply anesthetized with xylazine and ketamine cocktail and then injected intravenously via the tail vein with 1ug per gram body weight of ETX-594 and 1.6ul per gram body weight of Fluorescein labeled Griffonia (Bandeiraea) Simplicifolia Lectin I (FITC-BSL1, Vector Laboratories) for ten minutes. This dose and time point was chosen to insure saturation of intravascular ETX binding sites without causing overt tissue damage, respectively. Because animals were anesthetized and the short time point, no symptoms were observed. Animals were perfused with PBS via transcardiac perfusion until all the blood in liver had been cleared. Organs were harvested and embedded in Tissue-Tek O.C.T (Fisher Scientific) compound and frozen on dry ice. Organs were stored at -20°C until use. A cryostat was used to section tissue between 16–18um onto gelatin-coated slides and stored at -20°C until use. For peripheral tissue sections, sections were fixed in 4% PFA for 10 minutes at room temperature (RT) and then cover slipped with VECTASHIELD Antifade Mounting Medium with DAPI (Vector Laboratories). Interestingly, FITC-BSL1 did no bind to microvasculature of CNS organs when injected intravenously, indicating that the BSL1 receptor is not on the luminal surface of this vasculature. For CNS organs, sections were fixed in 4% PFA for 10 minutes at RT, washed with PBS, blocked in 10%FBS/PBS/0.1% triton-100 for 30 minutes, and then probed with FITC-BSL1 for 1 hour at RT. Sections were washed in PBS then cover slipped with VECTASHIELD Antifade Mounting Medium with DAPI. Alternatively, whole retinas and optic nerves were fixed overnight in 4% PFA at 4°C, washed in PBS, blocked in 10%FBS/PBS/0.1% triton-100 for 30 minutes at RT, probed with FTIC-BSL1 for one hour at RT, and then mounted on coverslips with VECTASHIELD Antifade Mounting Medium with DAPI. Tissues sections and whole mounts were imaged using Zeiss Axioskop2 Plus upright microscope (Oberkochen) and Spot RT3 camera and software (Spot Imaging). Images were post-processed using Adobe Photoshop (Adobe).

### *In vivo* BBB permeability assays

Wild type mice (C57BL/6J, The Jackson Laboratory), *Mal*-/- mice [82], and *Cav1*-/- mice (B6.Cg-Cav1tm1Mls/J, The Jackson Laboratory) were used in these experiments. Mice were treated with a lethal dose of 5ng of ETX (diluted in saline) per gram body weight, via intraperitoneal injection. In preliminary experiments, this dose was determined to cause moribund symptoms within one to three hours after treatment. We believe that this dose and time course better represent that “natural course” of ETX intoxication. Control mice received intraperitoneal injections of saline only.

To evaluate BBB permeability to fluorescein sodium salt (FITC-Na, Sigma Aldrich), mice were treated with ETX or the saline for one hour. After one hour, animals were intravenously injected with 60ug per gram body weight of FITC-Na via tail vein for ten minutes then euthanized with a lethal dose of xylazine and ketamine. Death occurred approximately 30 minutes after drug treatment. Brains were harvested, weighed, and homogenized in 1ml PBS by viscous vortexing with sterilized, glass beads. The brain homogenate was then centrifuged at 1500rcf for 10 minutes to pellet beads. Supernatant was removed and centrifuged at 18,000rcf for ten minutes to pellet cellular debris. The cleared supernatant from the homogenized brains was analyzed by spectrometry at 490nm. The amount of FITC-Na in supernatant homogenates was calculated using a FITC-Na standard curve diluted in PBS. Results were expressed as FITC-Na ug/ml per gram of brain. Results were normalized to genotype controls and expressed as FITC-Na Extravasation (% CT).

To evaluate BBB permeability to FITC labeled 70KDa dextran (Sigma Aldrich) control and ETX treated mice were observed for one hour after treatment and then intravenously injected via tail vein with 0.2mg per gram body weight of FITC-70KDa dextran for ten minutes. Animals were euthanized with lethal doses of zylazine/ketamine cocktail. Death occurred approximately 30 minutes after drug treatment. Organs were harvested and sectioned as previously descried. Previous studies have indicated that perfusion of animals after injection greatly underestimates that amount of tracer extraversion. In addition, some tracers, like dextran, are not fixable by PFA, so attempts to fix sectioned slides only causes tracers to be washed away [117–119]. As such, cryosections from mice who received 70KDA dextran injections were examined without any post processing or mounting media. Tissues were imaged using Zeiss Axioskop2 Plus upright microscope (Oberkochen) and Spot RT3 camera and software (Spot Imaging). Images were post-processed and were patched together into a composite image.

To evaluate BBB permeability using endogenous IgG as a marker, mice were treated with ETX or saline for three hours and perfused via transcardiac perfusion with PBS. Organs were harvested and sectioned as previously described. Tissue sections were fixed in 4% PFA for ten minutes at RT and then blocked in 10%FBS/PBS/0.1% triton-100 for 30 minutes at RT. Sections were probed with Cy3 conjugated anti-mouse IgG (Jackson ImmunoResearch) and FITC-BSL1 for 1 hour at RT. Sections were mounted on coverslips with VECTASHIELD Antifade Mounting Medium with DAPI (Vectorlabs). Tissues were imaged using Zeiss Axioskop2 Plus upright microscope (Oberkochen) and Spot RT3 camera and software (Spot Imaging). For figure micrographs, images were post-processed using Adobe Photoshop (Adobe). Saggital sections were used to measure endogenous IgG extravasation. Images were captured in the myelinated region of the cerebral cortex, superior to the hippocampus and lateral ventricle for all mice. Analyzed images spanned from the corpus callosum to subcortical gray matter. A minimum of three sections per mouse was captured. For relative fluorescence measurements, images were imported into ImageJ64 and converted into an 8-bit gray format. The threshold was identically adjusted for all IgG images and converted to a binary image. The mean and integrated density was calculated for each individual image and was used as a measurement of fluorescence density. The mean fluorescent density for each individual mouse was calculated and used to calculate the mean for an entire treatment group. Results were normalized to genotype controls and expressed as Ig Extravasation (% CT).

To evaluate BBB permeability to Alexaflour-594 conjugated bovine serum albumin (BSA-594, Molecular Probes), mice were treated with 5ng of ETX per gram body weight for 90 minutes via tail vein injection. Mice were then intravenously injected via tail vein with 300ug of BSA-594 for 30 minutes before being anesthetized with xylazine and ketamine and perfused with PBS followed by 4% PFA via trans cardiac perfusion. Sections were blocked in 10%FBS/PBS/0.1% triton-100 for 30 minutes at RT. Brain sections were probed with the pericyte marker anti-PDGFR-Beta (Abcam) for 1 hour at RT, washed with PBS, and then probed with DyLight conjugated anti-rabbit IgG (Jackson ImmunoResearch) for 1 hour at RT. Whole retinas were blocked in 10%FBS/PBS/0.1% triton-100 for 30 minutes at RT. Whole mounts were then probed with FITC-BSL1 for 1 hour at RT. Stained tissue sections or whole mounts were mounted on coverslips with VECTASHIELD Antifade Mounting Medium with DAPI. Tissues were imaged using Zeiss Axioskop2 Plus upright microscope (Oberkochen) and Spot RT3 camera and software (Spot Imaging). Images were post-processed using Adobe Photoshop (Adobe).

### Detection of ETX binding in *Cav1*+/+ and *Cav1*-/- mice

16–18uM brain sections form Wild type mice (Stain C57BL/6J, The Jackson Laboratory) or *Cav1*-/- mice (B6.Cg-Cav1tm1Mls/J, The Jackson Laboratory), were fixed for 10 minutes at RT in 4% PFA, washed with PBS, and blocked in 10%FBS/PBS/0.1% triton-100 for 30 minutes at RT. Sections were probed with 5ug/ml of proETX (BEI) for 1 hour at RT, washed with PBS, washed with PBS, and proETX binding detected using an affinity purified rabbit polyclonal antibody for 1 hour at RT[97]. Sections were washed again with PBS, and probed with Cy3 conjugated anti-rabbit IgG (Jackson Immunoresearch, 1:500) and FITC-BSL1 (Vector Laboratories, 1:200) for 1 hour at RT. Sections were mounted on coverslips with VECTASHIELD Antifade Mounting Medium with DAPI (Vector Labratories). Tissues were imaged using Zeiss Axioskop2 Plus upright microscope (Oberkochen) and Spot RT3 camera and software (Spot Imaging). For figure micrographs, images were post-processed using Adobe Photoshop (Adobe). To determine relative florescence of proETX binding to microvasculature of *Cav1*+/+ vs. *Cav1*-/- mice, at least four sections for each genotype were evaluated. Images from cerebellum gray matter tracts were imported into ImageJ64 and converted into an 8-bit gray format. The threshold was identically adjusted for all proETX and BSL1 images and converted to binary images. The mean and integrated density for BSL1 and proETX was calculated for each individual image and was used as a measurement of fluorescence density. proETX fluorescence intensity was normalized to BSL1 fluorescence intensity to determine relative fluorescence (pETX/BSl1) for each imaged brain section.

### Immunohistochemistry

For staining with anti-ZO1 (Invitrogen 1:20), VE-cadherin (Abcam, 1:200), or CAV1 (Abcam, 1:500); 16–18um brain sections were fixed for 10 minutes in 4% PFA and washed with PBS. Tissue was blocked and permeabilized in blocking buffer 5% FBS/PBS 0.1% triton-100 for 30 minutes at RT and washed with PBS. Primary antibodies were diluted in antibody diluent (5% FBS/PBS) and tissue sections probed for 1 hour at RT or ON at 4°C and washed with PBS. Tissue was then probed with an appropriate Cy3-conjugated secondary antibody (Jackson immunoresearch, 1:500) and FITC-BSL1 (Vector Laboratories, 1:200) for 1 hour at RT. Finally, slides were washed with PBS. Alternatively, tissue stained for claudin-5 (Abcam, 1:500) were fixed in methanol for two minutes prior to blocking and staining. All sections were mounted on coverslips with VECTASHIELD Antifade Mounting Medium with DAPI (Vector Laboratories). Tissues were imaged using Zeiss Axioskop2 Plus upright microscope (Oberkochen) and Spot RT3 camera and software (Spot Imaging) and post-processed using Adobe Photoshop (Adobe).

### Primary brain endothelial cell isolation and culture

Primary mouse brain endothelial cells (BEC) were isolated as previous described [120]. Brain microvessels were isolated from TIE2-GFP mice (Tg(TIE2GFP)287Sato/J, The Jackson Laboratory) to qualify endothelial cell purity unless otherwise noted. Briefly, whole brains were digested using the Papain Cell Dissociation System with DNase I (Worthington Biochemical). Microvessels vessels were isolated via centrifugation and plated on Collagen Type I (Millipore) coated 6-well, culture treated dishes. For imaging experiments, microvessels were seeded into 6 well plates containing four Poly-D-Lysine/Laminin Cellware 12mm round coverslips (Corning BioCoat) coated with Collagen Type I (Millipore). Microvessels were allowed to adhere to plates in endothelia cell growth medium (ECGM) composed of of F-12 (Gibco) stock medium containing 10% FBS, GlutaMAX, penicillin/streptomycin, 30ug/ml endothelia cell growth supplement (Millipore), 2.5ug/ml ascorbate, and 40ug/ml heparin. Cells were allowed to adhere for one day in a humidified 37°C incubator with 5% CO2, and then treated for two days with ECGM containing 4ug/ml puromycin to prevent growth of non-endothelial cells. Cells were allowed to reach confluence in ECGM without puromycin, which typically took 5 to 10 days. In some experiments, cells were trypsinized and seeded into collagen (3.48mg/ml) laminin (1mg/ml), and fibronectin (1mg/ml) coated-96 tissue culture treated plates. Cells were used at either passage zero (grown directly from microvessels) or passage 1 (grown in 96-well plates). BEC were treated with indicated ETX doses at indicated time points.

### Immunocytochemistry (ICC)

BEC grown on coverslips stained with anti-CAV1 (Abcam, 1:500), anti-EEA (Cell Signaling Technology, 1:100), anti-RAB5 (Cell Signaling Technology, 1:200), anti-RAB7 (Cell Signaling Technology, 1:100), anti-RAB11 (Cell Signaling Technology, 1:100), or anti-LAMP1 (Abcam 1:100) were fixed in 4% PFA for ten minutes at RT and washed with PBS. When stained with anti-claudin-5 (Abcam, 1:500, BEC were fixed in methanol for 2 minutes at RT. Fixed cells were blocked in 10% FBS/PBS/0.1% triton-100 for 30 minutes at RT and then probed with primary antibodies for 1 hour at RT or at 4°C overnight. Cells were then washed in PBS, probed with appropriate conjugated secondary antibody for 1 hour at RT, and mounted with VECTASHIELD Antifade Mounting Medium with DAPI (Vector Laboratories). Tissues were imaged using Zeiss Axioskop2 Plus upright microscope (Oberkochen) and Spot RT3 camera and software (Spot Imaging) and post-processed using Adobe Photoshop (Adobe). For relative fluorescence measurements, images were imported into ImageJ64 and converted into an 8-bit gray format. The threshold was identically adjusted for all anti-ETX images and converted to a binary image. The mean and integrated density was calculated for each individual image and was used as a measurement of fluorescence density. In addition, the number of nuclei per field were analyzed using the Analyze Particles function. Fluorescent density and nuclei count were exported to Microsoft Excel and the relative fluorescent density calculated by dividing the fluorescent density by the number of nuclei per field.

### Vacuolation analysis

Percent vacuolation was determined in fixed BEC on cover slips or in 96 well plates. For BEC on coverslips, DIC images were captured using Zeiss Axioskop2 Plus upright microscope (Oberkochen) and Spot RT3 camera and software (Spot Imaging). For 96 well plates, live DIC images were captured with an inverted fluorescence microscope (Nikon) equipped with a Charged Coupled Device (CCD) camera (Carl Zeiss) imaged with Spot software. The total number of BEC per field were identified by their large nuclei and counted manually with the use of the count tool in Adobe Photoshop (Adobe). Cells were determined to be vacuolated if they contained two or more, phase-bright vacuoles. The percent vacuolated was calculated by dividing the number of cells with vacuoles by the total number of cells. In some experiments, 50ug/ml of propidium iodine (PI, Sigma Aldrich) were added to wells to determine cell death. Cells were considered dead if nuclei were PI positive. For inhibition of vacuolation, BEC were pretreated with 20uM of nocodazole (Sigma Aldrich), 20uM of MiTMAB (Abcam), 25uM Pitstop 2 (Abcam), or 5ug/ml of filipin (Sigma Aldrich) for 30 minutes at 37°C. Untreated cells were used as controls. Cells were then treated with or without 50nM ETX for 2 hours. The percent of vacuolated cells was determined for each treatment. To determine the percent of vacuolated cells per pretreatment group, the percent of vacuolated cells in each pretreatment group was divided by the percent of vacuolated cells treated with ETX alone and expressed as % ETX alone.

### Cell death analysis

To determine the percentage of dead cells, BEC in 6 well dishes were treated with indicated doses of ETX for four hours and a single cell suspension achieved by trypsinization. Trypsinization was stopped by adding a surplus of FBS to resuspended cells. Cell death was analyzed by propidium iodide (PI, Sigma Aldrich) inclusion assay by treating resuspended cells with 2ug/ml of PI and analyzed using a BD FACSVerse Flow Cytometer. Data was collected using FACSuite software and analyzed using FlowJo software. In 96-well experiments, BEC were treated with the indicated amount of ETX for the indicated timepoints and treated with 50 μg/mL of PI. Live images of randomly chosen fields in each well were acquired under an inverted fluorescence microscope (Nikon, Minato, Tokyo, Japan) equipped with a Charged Coupled Device (CCD) camera (Carl Zeiss, Oberkochen, Germany) imaged with Spot software and were then imported into ImageJ64 in 8-bit gray format. For quantification of PI-positive cells, the images were converted into binary images by applying the same threshold value to all images collected from the same experiment. Analyze Particles function was selected to automatically count the particle numbers and to analyze particle properties, such as size shape, and distribution patterns. Data were exported and analyzed in Excel. Results were normalized to untreated controls and expressed as Cell Death (% of CT).

### Scanning electron microscopy

Control and ETX treated BEC grown on coverslips were fixed in a fixative, containing 2% paraformaldehyde and 2.5% glutaraldehyde in 0.075M sodium cacodylate buffer pH 7.4. Tissue were dehydrated by a graded series of ethanol (50%, 75%, 95%, and 100% 3 times) and followed by a critical point dry (CPD), which was initiated in the ethanol-filled chamber and replaced with carbon dioxide liquid in the CPD chamber (Autosamdri A-815, Tousimis). Five nm thickness iridium to coating was applied over the dried samples (ACE600, Leica). The samples were examined under a SEM (LEO 1550; Carl Zeiss) with a field-emission electron gun and operation/data acquisition software (Smart SEM version 5). EM study was conducted at the Rockefeller University Electron Microscopy Resource Center.

### Transmission electron microscopy

BEC in 6 well plates were washed in serum free medium and fixed in 2.5% glutaraldehyde, 4% paraformaldehyde, 0.02% picric acid in 0.1M Na-cacodylate buffer for 30 mins at RT or 4C overnight. Cells were washed three times with 0.1M Na-cacodylate and secondary fixed in 1:1 1% OsO4–1.5%K-ferricyanide (aqueous) for 60 mins. Cell were washed three times in 1m Na–Cacodylate and once in deonixed water. Cells were incubated in 1.5% Uranyl Acetate for 1 hour in the dark and dehydrated for1 0–15 mins in ethanol in increasing concentrations. Cells were washed in LX-122 resin to remove any leftover ethanol and then fully immersed in resin. Bottomless and topless BEEM capsules were used to capture cells in resin and were cured at 50°C overnight. Samples were allowed to polymerize for 1.5 days and then sectioned using a diamond knife (Diatome, US, Hatfield, PA) mounted on a Leica Utracut T ultramicrotome at a section thickness of 65nm. Images were captures using TEM microscope JEOL JSM 1400, operated at 100Kv. Images were collected using a Veleta 2k x 2k CCD camera (EM-SIS, Germany). To measure the number of caveolae per um of cell surface, TEM images from vertical BEC sections were imported into ImageJ64. The length of the cell surface of overlapping, sequential images was measured using the segmented line tool. The scale bar generated by the imaging software was used to set the ImageJ64 scale used to determine the actual cell surface length. The number of caveolae were manually counted and expressed as number of caveolae/um. Over 200um of apical cell surface was evaluated for each replicate.

### Protein extraction and western blot analysis

BEC cells grown to confluence in 6 dishes were treated with indicated doses of ETX in 2ml of media containing 50nM ETX for the indicated time at 37 °C in 5% CO2 in a humidified incubator. Cells were moved to ice, then washed three times with ice cold PBS. Cells were lysed in 1ml ice-cold ULTRARIPA A Buffer (BioDynamics Laboratory Inc) containing proteinase and phosphatase inhibitors (Cell Signaling Technologies) for 10 min. Lysates were centrifuged at 5000rcf to pellet DNA and the was supernatant used for analysis. All samples were prepared in 2X Laemmli Sample Buffer (Bio-Rad, Hercules, CA, USA) containing 5% 2-Mercaptoethanol (Bio-Rad) and heated at 95°C for 5 min before loading onto 4–20% Mini-PROTEAN TGX Stain-Free gels (Bio-Rad). Gels were run in Tris/Glycine SDS Buffer (Bio-Rad) at 200 V for 25 min. Semi-dry transfers were performed in transfer Tris/Glycine Buffer (Bio-Rad) using the Trans-Blot SD Semi-Dry Electrophoretic Transfer Cell system (Bio-Rad) at 15 V for 15 min. Blots were blocked in 5% Blotting-Grade Blocker nonfat milk (Bio-Rad) in Tris Buffered Saline with Tween 20 (TBS-T, Cell Signaling Technology) for one hour at room temperature. Blots were then incubated with anti-ETX antibody JL004[97] at 0.34 μg/mL in blocking solution for 1 hour at RT. Blots were washed with TBS-T at room temperature and incubated with secondary antibody peroxidase-conjugated Affinipure Goat Anti-Rabbit IgG H + L (Jackson ImmunoResearch) at 0.024 μg/mL in blocking solution for 1 hour at RT. Blots were washed again in TBS-T and developed for 5 min at room temperature in SuperSignal West Dura Extended Duration Substrate (ThermoFisher Scientific). The developed blots were visualized on 5x7 CL-XPosure Films (ThermoFisher Scientific) at various exposure times using a Konica Minolta SRX-101A film processor. Densitometry measurements were taken from scanned films using ImageJ64 software (National Institutes of Health).

## Supporting information

S1 Fig Haemotoxylin and eosin staining of selected organs from saline and ETX treated mice. Mice were injected via IP with 5ng of active ETX per gram of body weight; saline treated animals were used as controls. Mice were euthanized with CO2. Following gross examination, all organs were fixed in 10% neutral buffered formalin, followed by decalcification of bone in a formic acid solution (Surgipath Decalcifier I, Leica Biosystems). Tissues were then processed in ethanol and xylene and embedded in paraffin in a Leica ASP6025 tissue processor. Paraffin blocks were sectioned at 5 microns, stained with hematoxylin and eosin (H&E), and examined by a board-certified veterinary pathologist. The following tissues were processed and examined: heart, thymus, lungs, liver, gallbladder, kidneys, pancreas, stomach, duodenum, jejunum, ileum, cecum, colon, lymph nodes (submandibular, mesenteric), salivary glands, skin (trunk and head), urinary bladder, uterus, cervix, vagina, ovaries, oviducts, adrenal glands, spleen, thyroid gland, esophagus, trachea, spinal cord, vertebrae, sternum, femur, tibia, stifle join, skeletal muscle, nerves, skull, nasal cavity, oral cavity, teeth, ears, eyes, pituitary gland, brain. Light microscopic examination did not reveal any significant differences between the two treatment groups at this timepoint and dose. Representative images from brain, heart, lung, and intestines from control and ETX treated mice are displayed. Scale bar is 200um.
(TIF)

S2 Fig Evaluation of lysosomes and endosomes in ETX treated BEC. (A) BEC were treated with or without 50nM ETX for 4 hours and then stained with Cytopainter Lysosomal Staining Kit (Abcam, ab112137) per the manufactures instructions. Live images were taken as described in methods section. (B) Fluorescent measurement of lysosmal staining from BEC treated with or without 50nM ETX for 4 hours. Results expressed as mean ± SEM, n = 3, p = 0.88 determined by T-Test. ICC staining for RAB5 (C) or RAB11 (D) of BEC treated with our without 50nM ETX for 2 hours as described in methods sections.
(TIF)
