# Supplementary figures and images for: Clostridium perfringens epsilon toxin induces blood brain barrier permeability via caveolae-dependent transcytosis and requires expression of MAL
Source: PLoS Pathog. 2019 Nov 8;15(11):e1008014. doi: 10.1371/journal.ppat.1008014 (PMC6867657; doi:10.1371/journal.ppat.1008014)

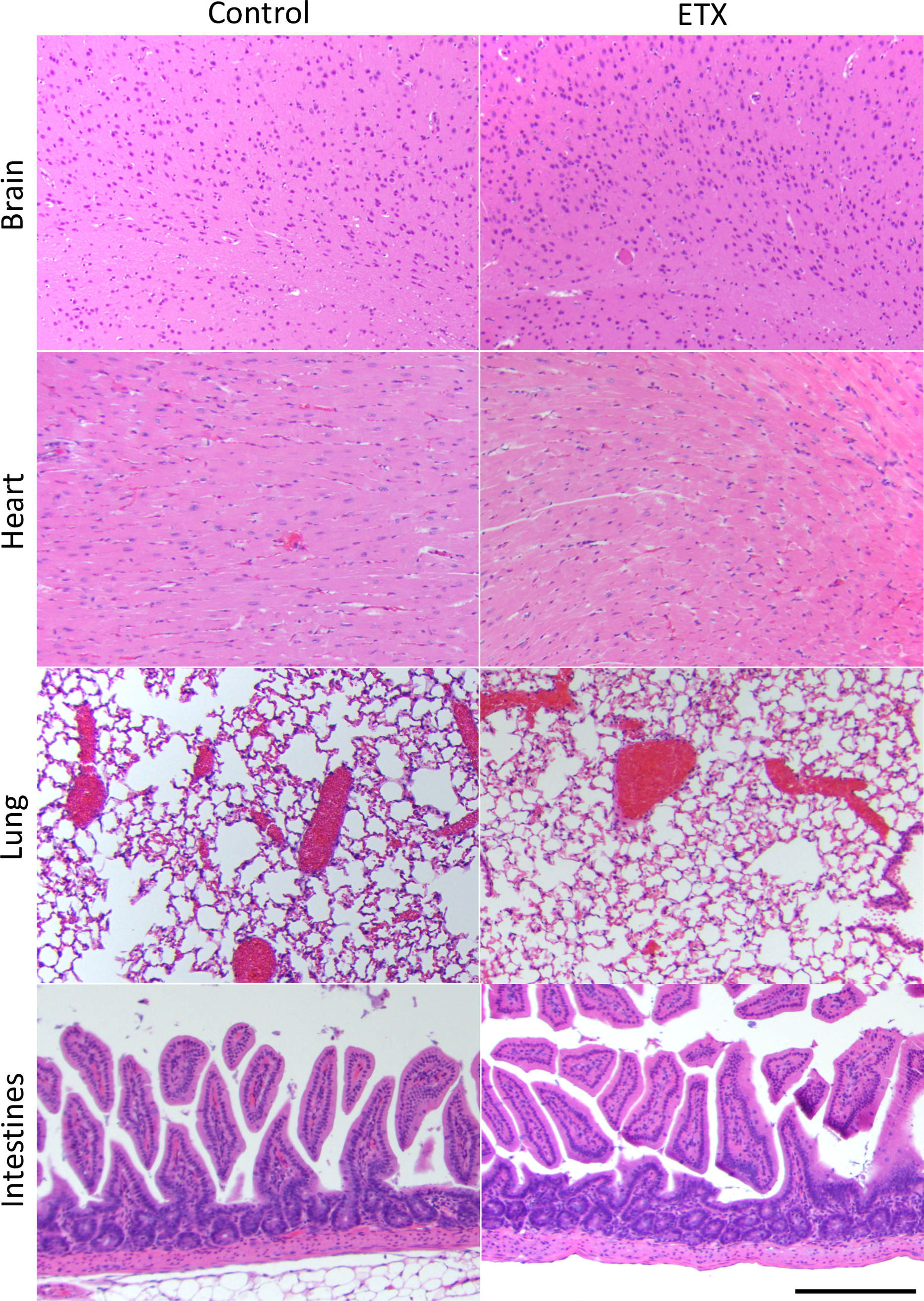

Supplement: S1 Fig — Mice were injected via IP with 5ng of active ETX per gram of body weight; saline treated animals were used as controls. Mice were euthanized with CO2. Following gross examination, all organs were fixed in 10% neutral buffered formalin, followed by decalcification of bone in a formic acid solution (Surgipath Decalcifier I, Leica Biosystems). Tissues were then processed in ethanol and xylene and embedded in paraffin in a Leica ASP6025 tissue processor. Paraffin blocks were sectioned at 5 microns, stained with hematoxylin and eosin (H&E), and examined by a board-certified veterinary pathologist. The following tissues were processed and examined: heart, thymus, lungs, liver, gallbladder, kidneys, pancreas, stomach, duodenum, jejunum, ileum, cecum, colon, lymph nodes (submandibular, mesenteric), salivary glands, skin (trunk and head), urinary bladder, uterus, cervix, vagina, ovaries, oviducts, adrenal glands, spleen, thyroid gland, esophagus, trachea, spinal cord, vertebrae, sternum, femur, tibia, stifle join, skeletal muscle, nerves, skull, nasal cavity, oral cavity, teeth, ears, eyes, pituitary gland, brain. Light microscopic examination did not reveal any significant differences between the two treatment groups at this timepoint and dose. Representative images from brain, heart, lung, and intestines from control and ETX treated mice are displayed. Scale bar is 200um. (TIF) [file ppat.1008014.s001.tif]

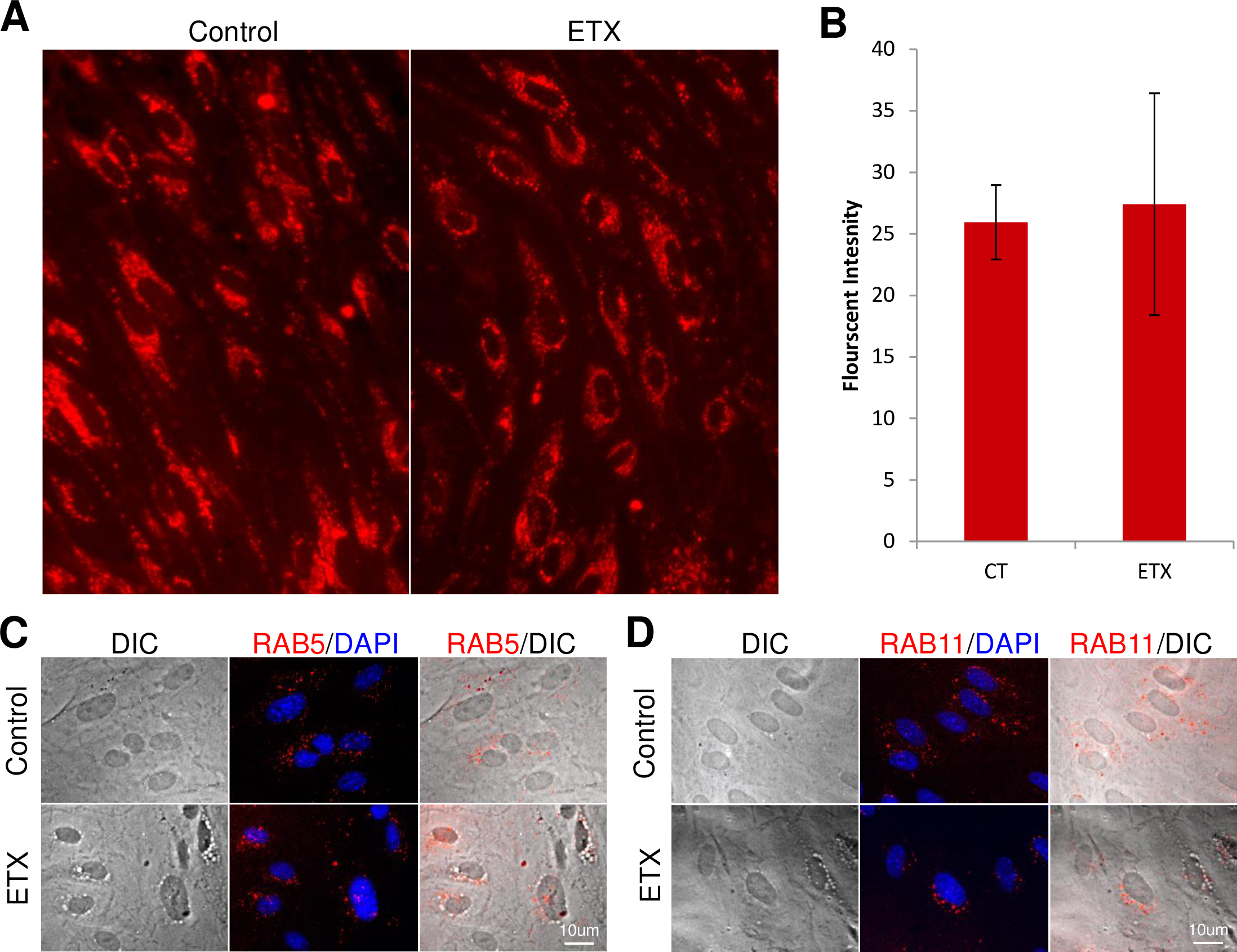

Supplement: S2 Fig — (A) BEC were treated with or without 50nM ETX for 4 hours and then stained with Cytopainter Lysosomal Staining Kit (Abcam, ab112137) per the manufactures instructions. Live images were taken as described in methods section. (B) Fluorescent measurement of lysosmal staining from BEC treated with or without 50nM ETX for 4 hours. Results expressed as mean ± SEM, n = 3, p = 0.88 determined by T-Test. ICC staining for RAB5 (C) or RAB11 (D) of BEC treated with our without 50nM ETX for 2 hours as described in methods sections. (TIF) [file ppat.1008014.s002.tif]
